# A Bibliometric Analysis of Research on Selenium in Drinking Water during the 1990–2021 Period: Treatment Options for Selenium Removal

**DOI:** 10.3390/ijerph19105834

**Published:** 2022-05-11

**Authors:** Ricardo Abejón

**Affiliations:** Departamento de Ingeniería Química, Universidad de Santiago de Chile (USACH), Av. Libertador Bernardo O’Higgins 3363, Estación Central, Santiago 9170019, Chile; ricardo.abejon@usach.cl

**Keywords:** selenium, drinking water, treatments, bibliometric analysis, research trends

## Abstract

A bibliometric analysis based on the Scopus database was carried out to summarize the global research related to selenium in drinking water from 1990 to 2021 and identify the quantitative characteristics of the research in this period. The results from the analysis revealed that the number of accumulated publications followed a quadratic growth, which confirmed the relevance this research topic is gaining during the last years. High research efforts have been invested to define safe selenium content in drinking water, since the insufficient or excessive intake of selenium and the corresponding effects on human health are only separated by a narrow margin. Some important research features of the four main technologies most frequently used to remove selenium from drinking water (coagulation, flocculation and precipitation followed by filtration; adsorption and ion exchange; membrane-based processes and biological treatments) were compiled in this work. Although the search of technological options to remove selenium from drinking water is less intensive than the search of solutions to reduce and eliminate the presence of other pollutants, adsorption was the alternative that has received the most attention according to the research trends during the studied period, followed by membrane technologies, while biological methods require further research efforts to promote their implementation.

## 1. Introduction

Selenium (Se), with atomic number 34, is a member of group 16 of the periodic table and thus belongs to the chalcogens. The position between the nonmetal sulfur and the metalloid tellurium determines the mainly nonmetallic properties it presents, characterized by high chemical similarity to sulfur. Because of its applications (electronic components, glass additives, metal alloys, etc.) and influence on human and animal health, research related to selenium has gained attention and issues about environmental pollution have become relevant [1].

The speciation of selenium in the natural environment is a key aspect to understanding its mobility, availability and toxicity. This nonmetal can be stable in several oxidation states but the most important ones from the environmental point of view are Se^−2^, Se^0^, Se^+4^ and Se^+6^. Elemental selenium, Se^0^, normally exists in the hexagonal semimetallic form (gray selenium) at ordinary temperatures, although other allotropic forms, mainly as red monoclinic selenium and different amorphous solids (black and red), can be found [2]. Nevertheless, it rarely occurs in its elemental native state or as pure ore compounds in the Earth’s crust. The Se^−2^ valence (selenide) is not frequent in aquatic environments, since it is only present under extreme redox circumstances. The Se^−2^ system includes H_2_Se and the corresponding deprotonated derivatives HSe^−^ and Se^−2^ with dissociation constant values of 3.8 and 14.0 for pK_a1_ and pK_a2_, respectively [3]. Although reduction of other selenium compounds, including insoluble elemental selenium, to selenide may occur due to microbial action [4], reaction between the dissolved selenide and metallic cations present in natural waters takes place, which results in the precipitation of insoluble metal selenides [5].

Therefore, the two most common oxidation states of selenium in water are Se^+4^ and Se^+6^ as part of the dissolved oxyanions selenite (SeO_3_^−2^) and selenate (SeO_4_^−2^), respectively [6]. Both species can be present in different protonated forms as function of the pH. On the one hand, the Se^+4^ system includes H_2_SeO_3_ and the corresponding deprotonated derivatives HSeO_3_^–^ and SeO_3_^−2^ with dissociation constant values of 2.7 and 8.5 for pK_a1_ and pK_a2_, respectively. On the other hand, the Se^+6^ system includes H_2_SeO_4_ and the corresponding deprotonated derivatives HSeO_4_^−^ and SeO_4_^−2^, with dissociation constant values of −2.0 and 1.8 for pK_a1_ and pK_a2_ [3]. According to these data, the prevalent species around neutral conditions (typical pH for natural surface waters and groundwaters ranges from 6.5 to 8.5) are HSeO_3_^−^ (maybe SeO_3_^−2^ when the pH is in the upper range) for Se^+4^ and SeO_4_^−2^ for Se^+6^. This fact implies that both valences remain as anions in most water bodies, since the highest pH value compatible with a non-charged molecule is lower than 3 (possible presence of H_2_SeO_3_).

**Figure 1:**
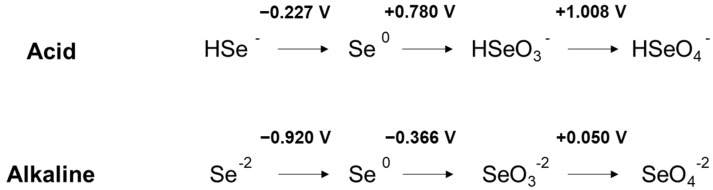
The redox potentials of selenium in acid and alkaline solutions.

In addition to pH, the redox potential also plays a relevant role in the definition of the relative abundance of the selenium species. Complete speciation diagrams for selenium in aqueous systems as function of pH and redox potential can be found in bibliography [7,8]. On one hand, under oxidant conditions, the Se^+6^ state becomes clearly dominant over the Se^+4^ one, but, on the other hand, the Se^+4^ species are prevalent under reducing conditions. Nevertheless, in case of extreme reducing conditions, Se^−2^ valence will become dominant. To gain a clearer idea, the redox potentials of selenium in acid and alkaline solutions are included in Figure 1 [3].

Therefore, the selenate system is thermodynamically more stable for surface waters under alkaline conditions, while in acidic waters selenite is predominant. Although selenite in these acid solutions could be reduced at least partially to insoluble elemental selenium under suitable redox conditions, complete removal is often difficult, because the selenium sometimes precipitates as a colloid and further reduction to selenide is very slow [9]. The case of groundwaters is a bit more complex, since both selenite and selenate states can coexist (even selenide can appear under reducing conditions) and the incidence of each specie depends on the total selenium input to the system, the specific chemical conditions and the biological activity.

The effects of selenium on human health have been subject to extensive research. Selenium plays a vital role in different physiological processes and its altered levels have direct impact on human health, since they can be directly related to the development of diseases [10]. Selenium is an essential micronutrient for humans and other animals, since it is important for many cellular processes because it is a component of several selenoproteins and selenoenzymes, such as glutathione peroxidase, with essential biological functions [11]. The biological activity of these selenium biological compounds is mainly related to antioxidant actions, activation and degradation of thyroid hormones and immunity enhancement [12]. Further detailed information about the role of selenium and its functions in the human body can be consulted [13].

Examples of health problems in farm animals caused by both excessive exposure (selenium toxicity) and suboptimal intake (selenium deficiency) have been well-known and the possible impact on human health of these situations has gained great concern [6]. On the one hand, excessive low intake of selenium in humans is directly related to the development of two endemic diseases that mainly occur in China and adjacent countries: a fatal dilated cardiomyopathy called Keshan disease [14,15,16] and a disabling degenerative disorder of peripheral joints and spine called Kashin–Beck disease [17,18,19,20]. On the other hand, a chronic high selenium intake by humans results in selenosis, characterized by symptoms such as hair and fingernails loss, diarrhea, effects on the central nervous system, loss of appetite and hepatic disfunction [21,22,23]. In addition, early symptoms of acute selenium poisoning include hypotension and tachycardia, vomiting, abdominal pain or diarrhea and neurological signs, such as tremor, muscle spasms, restlessness and confusion. Pulmonary edema develops as a severe complication and in severe cases, death can be reached due to peripheral vasodilatation or direct myocardial depression [24,25].

Consequently, controlled dietary intake of selenium is highly recommended. The World Health Organization (WHO) established the limits for recommended selenium intakes between 25 and 35 μg/d, depending on the genre, with even lower values for infants, children and adolescents [26]. This recommendation clearly reduced previously defined dietary limits, with typical values above 50 μg/d [27]. The uptake and accumulation of selenium by plants define the transference of this element from soils to animals, including humans. Different plant species have different abilities to take selenium from soil, and different plant tissues differ in their selenium contents [28,29]. The bioaccumulation of selenium in food chain components across trophic levels has been investigated for different ecosystems [30,31]. Therefore, the content of Se in different diets varies significantly as a function of both soil and plant and animal species. In addition to food sources, drinking water must be taken into account as a significant source of selenium intake, specifically in regions with selenium-rich soils or waters [32,33].

The simultaneous essentiality and toxicity of selenium for humans have created a great controversy about safe limit values for selenium in drinking water. This debate is not new, since it started in the 1970s and early 1980s, with the scientific discussion related to the justification of a new recommendation of 50 µg/L in the United States, versus the originally proposed 10 µg/L concentration for selenium in drinking water [34,35,36]. The WHO produces its international norms on water quality and human health in the form of guidelines that are used as the basis for regulation and standard setting. For the particular case of selenium, the standard limit was fixed at 10 µg/L until it was increased to 40 µg/L in 2011 when the fourth edition of the guideline was published [37]. Nevertheless, most jurisdictions nowadays continue applying a threshold value of 10 µg/L in their corresponding legislations [38], including Chile [39] and the European Union. In this last case, even the proposal approved in 2018 to review the European Directive justified the maintenance of the 10 µg/L limit against the new recommended value by the WHO [40]. However, the adopted final Directive defined a 20 µg/L limit, which can be increased until 30 µg/L for regions where geological conditions could lead to high levels of selenium in groundwater [41]. Nevertheless, scientific researchers continue the discussion and propose new limit values below and above the 10 µg/L concentration [42,43,44].

The presence of selenium in the environment has a highly irregular distribution among the atmospheric, aquatic and terrestrial compartments. The latter one is the most relevant compartment, but natural processes can transfer selenium to groundwaters and surface waterbodies, such as volcanic activity; rock and soil weathering; leaching of soils; transportation by groundwater; uptake and release by plants, animals and microorganisms; adsorption-desorption reactions; or chemically and biologically mediated oxidation-reduction reactions [45]. Although the selenium content of most natural waters does not threaten human health, the aquifers and the related surface water bodies in natural selenium-rich geological areas can present selenium concentrations that require further treatment to obtain safe drinking water. Chinese, Indian, American and Canadian selenium-rich regions have been deeply investigated [46,47,48], but other countries with localized areas characterized by high selenium contents can be mentioned, such as Argentina, Brazil, France, Ireland, Israel, Italy or Venezuela [49,50,51,52,53,54,55,56]. Nevertheless, anthropogenic activities account for a widespread selenium contamination as the result of some industrial activities, such as coal mining and combustion; gold, silver and nickel mining; metal smelting (especially pyrometallurgical copper, nickel and zinc production); oil transport, refining and utilization; and agricultural irrigation with selenium-rich waters [57]. Examples of many locations where waterbodies have been polluted by these industrial activities have been deeply identified and investigated [58,59,60,61,62,63,64,65,66,67,68,69,70], including the case of Chile, where samples of drinking water with selenium concentration above 10 µg/L have been analyzed [71]. Since no natural geological area rich in selenium has been highlighted in Chile [72,73], the presence of selenium in drinking water can be directly related to the copper mining, smelting and refining activities in most cases [74,75,76].

Since the management of the high number of published papers about selenium and drinking water that can be found in bibliography is difficult, bibliometric tools are useful to handle all this information. Bibliometrics refers to the research methodology employed in library and information sciences, which applies quantitative analysis and statistics methods to describe the distribution patterns of publications according to some given categories. This methodological approach allows the exploration, organization and analysis of a high number of scientific documents and can be applied to the identification of important research trends, as demonstrated by several works in the environmental and chemical engineering fields [77,78,79,80,81,82,83,84,85,86,87,88,89,90,91,92], including water pollution aspects [93,94,95,96,97,98,99,100,101,102,103].

The main purpose of this work was to analyze, from a bibliometric perspective, the scientific literature related to the research on selenium in drinking water published from 1990 to 2021 in the sources compiled in Scopus. These documents were analyzed and evaluated according to several categories (annual outputs, leading countries and institutions, or main journals, subjects and languages) and were used to determine the quantitative characteristics of the research on selenium removal from drinking water worldwide. In addition, a bibliometric network analysis was carried out to contribute to the identification of the most relevant trends related to this topic and possible research gaps.

## 2. Data Sources and Methodology

The bibliographic search of published scientific literature related to selenium in drinking water was based on the employment of Scopus database. This abstract and indexing database with full-text links is managed by Elsevier and claims to index over 22,800 active titles from more than 5000 international publishers. These figures imply that it is the largest abstract and citation database of peer-reviewed literature and delivers the most comprehensive view of the world’s research output in the fields of science and technology [104]. More than 69 million abstracts with references back to 1969 and more than 6 million records before that year are included. Titles from all regions around the world are covered, counting non-English titles when abstracts in English are provided with the documents. In fact, around 20% of titles on Scopus are not published in English, resulting in more than 40 languages. In addition, more than 50% of Scopus content comes from outside North America, with important contributions by European, Latin American and Asian countries. As a result, Scopus offers an extensive coverage of peer-reviewed literature across the sciences, technology, engineering and mathematics (STEM) fields.

The online search within Scopus was completed in April 2022 after the selection of “*selenium*” and “*drinking water*” as keywords in the Article Title, Abstract, Keywords field of the search-engine. The keywords *drinking* and *water* were introduced together with quotations to obtain only the papers that include these two words in the exact sequence. The search was limited from 1990 to 2021 in order to identify the scientific documents related to the research on this topic published before 2022. The total number of documents recovered was 1117.

The analysis of the scientific literature obtained after a systematic bibliographic search provides a suitable scenario to have a better understanding of the global research situation in such a relevant subject as removal of selenium from drinking water, which can support the identification of present hot topics and the definition of future long-term research strategies. Consequently, the investigated aspects included in this work did not only cover the quantitative description of the publications (annual outputs, leading countries and institutions, or main journals, subject categories and languages), but also the review of the most relevant research topics identified after the study of the corresponding keywords.

## 3. Results and Discussion

### 3.1. Bibliometric Analysis of Research Trends on Selenium in Drinking Water (1990–2021)

#### 3.1.1. Publication Year, Document Type and Language of Publications

**Figure 2:**
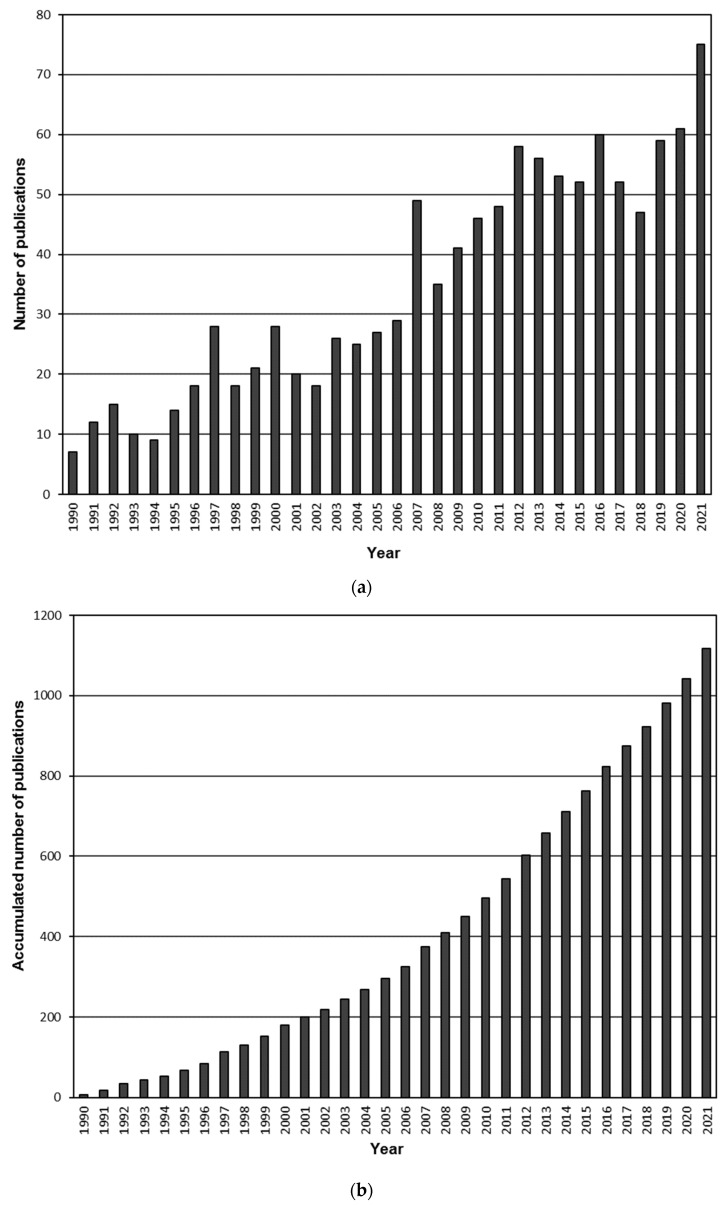
Annual (**a**) and accumulated (**b**) publication output.

The distribution of annual publication output identified by Scopus and the total number of accumulated documents are shown in Figure 2. It is obvious that there is a continuously increasing general trend in the number of publications that appears each year, although three different stages can be distinguished. The first one covers the 1990–2002 period and it is characterized by an irregular evolution of the number of publications, where the years 1997 and 2000 must be highlighted because of their prolific production. From 2002 to 2011 a much more regular linear increase can be identified but the year 2007 was especially productive and has the highest value in this period. After 2011, another irregular stage appeared, in this case with a great production rate maintained over time, since only 2018 did not attain 50 annual publications. As a consequence, the references published for the last ten years (from 2012 to 2021) account more than half of the total found publications during the 32-year period (51.3%). Nevertheless, when the accumulated number of publications was observed, the corresponding rise can be considered as a quadratic growth and it was decided to apply a quadratic regression to the data. The obtained equation was y = 0.995·x^2^ + 2.44·x + 17.4, where y represents the number of accumulated documents and x the year (starting at 1 for the year 1990). The result of the regression was a very good fitting, with a R^2^ value of 0.9991.

The distribution of document types was analyzed. Eleven different document types were found among the total 1117 publications. Nevertheless, article (967) was the most frequently used document type comprising 86.6% of total production, followed by review (65; 5.8% contribution) and proceedings paper (49; 4.4% contribution). These percentages, and specifically the clear supremacy of articles over other types of publication, are very concordant with the figures obtained by other authors when analyzing the trends on the research about other contaminants in water [93,100,105]. The other less significant categories include book chapter (16), note (7), editorial (5), short survey (3), letter (2), book (1), erratum (1) and retracted (1).

A clear majority (94.0%) of all the publications were published in English. Several other languages were identified, Chinese and Russian being the second and third languages, respectively. The rest of languages represented are compiled in Table 1. English has undoubtedly turned into the global lingua franca and there has never in the past been a language spoken more widely in the world than English is today [106]. Consequently, international communication has moved to a clear pre-eminence of English, especially in the field of scientific research, where more than 75% of the published documents in the social sciences and humanities and well over 90% in the natural sciences are written in English [107]. However, due to China’s fast development in research production and its high percentage of national journals published in Chinese, the world is experiencing, for the first time in more than a century, a decrease in the worldwide percentage of active academic journals published in English and an increase in the percentage of documents written in Chinese [108].

#### 3.1.2. Publication Distribution of Countries and Institutions

The top 31 countries (the only ones that produced at least 10 documents) ranked by number of total publications are shown in Table 2. Since the country affiliation is not an exclusive category (a document can be contributed by authors from more than one country), some papers may be indexed in more than one country simultaneously. Consequently, the sum of the number of documents in these categories is above the total number. A reduced group of countries usually dominate the global scientific production, as in this case, since the joint contribution of the three first countries in the ranking (USA, China and India) accounts for 47.3% of the total number of documents. USA is the most productive country, with 259 papers, which implies a percentage of 23.2%. This leader country was followed by two Asian countries (China and India) which jointly produce a percentage higher than the one corresponding to USA (24.7%). After Canada and Japan, the top ten positions are completed with European countries: among them Turkey is surprisingly the most prolific country with 44 documents, followed by Italy, Germany, the United Kingdom and France, which are countries with relevant contributions in most research fields. However, the presence of countries with limited scientific production in other topics has been previously identified by other bibliometric studies regarding pollution of drinking water [93]. This fact was explained by the relative importance of the presence of polluted drinking water in these parts of the world and some countries, such as Tunisia, Bangladesh, Egypt or Nigeria, which are included in Table 2, could be mentioned as examples of countries worried by the presence of selenium in drinking water [109,110,111,112].

In fact, these countries worried by the presence of polluted waters have deserving contributions when additional indicators that give the possibility of having some benchmarking are analyzed. Besides the total number of publications, two other indicators that take into account the total population and income (GPD) of the countries have been considered in Table 2: the number of publications per million inhabitants and the number of publications per trillion US Dollars (population and income data taken from World Bank database). On the one hand, when the income indicator is observed in detail, Tunisia appeared as the leader with a great difference, since it obtained a value above 600 document/trillion $, which is an order of magnitude higher than the following countries. In this ranking, the second, third and fourth positions corresponded to Bangladesh, Egypt and Pakistan, which are not high-income countries. A group of three European high-income countries occupied the next three positions: Greece, Sweden and Czech Republic in the fifth, sixth and seventh places, respectively. On the other hand, the analysis of the population indicator demonstrated the important research efforts promoted by Scandinavian countries, such as Sweden, Norway and Denmark, are situated in the first, second and fourth positions, respectively. Once again, Tunisia must be highlighted, since it occupied the third position in this ranking, with a contribution above two documents/million inhabitants, a limit only surpassed by four countries.

The top 18 institutions (the only ones with at least 10 documents) are compiled in Table 3. Among these top 18 institutions, 5 were in China and 4 in the USA, thus although USA was the most productive country, this production was shared more among different institutions. In the case of China, its production is more concentrated and the leader (Chinese Academy of Sciences with 40 documents) and the second (Institute of Geographical Sciences and Natural Resources Research with 19 documents) institutions were Chinese. Surprisingly, the third position was occupied by a Tunisian university (University of Sfax), which contributed with 16 documents, just two more than the production of the US EPA, a very relevant institution in all the topics related to water pollution. The role of the Panjab University in India, with 14 documents published, the same amount that US EPA, must be highlighted. Moreover, the great concern about the effects of selenium on human health justified the presence of prestigious medical institutions in the ranking, such as the Swedish Karolinska Institute, which is one of Europe’s largest and most prestigious medical institutions, or the Columbia Mailman School of Public Health in the USA.

#### 3.1.3. Distribution of Output in Subject Categories and Journals

The distribution of subject categories defined by Scopus is shown in Table 4, where the 9 most popular categories are compiled (the only ones with at least 40 articles), taking into consideration once again that some documents can be included in more than one subject, since it is not an exclusive category. The ranking indicates that *Environmental Science* was the most common subject, but the role of the biomedical sciences must be highlighted, since *Medicine* occupied the second position and the third position corresponded to *Biochemistry, Genetics* and *Molecular Biology.* Moreover, *Pharmacology, Toxicology and Pharmaceutics* occupied the fifth position of the ranking, just after *Chemistry* in the fourth position. These results are in agreement with those ones obtained by a bibliometric analysis applied to the research trends on lead in drinking water [100], but they do not fit as well with the trends identified in the case of a bibliometric analysis about arsenic in drinking water [93]. The *Engineering* category was very relevant and it occupied the second position in the ranking, while in this case, it occupied the eighth position (49 documents), just two publications more than chemical engineering, which ranked ninth with 47 documents. This fact gives a clear idea about the significant efforts applied to the search of effective technical solutions to the problems caused by the presence of arsenic in drinking water, but in the case of selenium, the efforts are more focused on the identification of the health effects of the intake of selenium.

The distribution of outputs in journals is shown in Table 5. The Scimago Journal Ranking indicator (SJR) of the top 9 journals, which are the only ones that published at least 15 articles, was also included. The two leading journals must be highlighted since their productions more than double the production of the journal in the third position. On the one hand, *Biological Trace Element Research* was the most prolific journal (54 documents) in articles related to selenium and drinking water. This journal is focused on the interdisciplinary field of research on the biological, environmental, and biomedical roles of trace elements. On the other hand, *Science of the Total Environment* occupied the second position in the ranking with 44 documents. It is a multi-disciplinary journal for publication of original research on the whole environment, which includes the atmosphere, hydrosphere, biosphere, lithosphere and anthroposphere. Therefore, these journals confirmed the relevance of the health and environmental aspects of the presence of selenium in the water bodies. A glance at the rest of the journals in the table is enough to discover the importance of the environmental studies regarding selenium, since all these journals contain the word environmental in their title, except the *Journal of Trace Elements in Medicine and Biology*, which covers biomedical issues related to trace elements. The most relevant journals according to the JCR indicators among the top journals are *Environmental Science and Technology* and *Environmental Health Perspectives*, both with JCR values above 2 (2.851 and 2.257, respectively). On the contrary, *Environmental Monitoring and Assessment* was the journal with the lowest JCR value, with a value below 0.6 (0.590).

#### 3.1.4. Most Frequently Cited Papers

The top 10 articles according to the number of citations they have received are presented in Table 6. The numbers of citations increased from 226 for the last article to 1278 for the leading article that occupied the first position of the ranking. In addition to the total number of citations, the Field-Weighted Citation Impact (FWCI) was also included. FWCI is the ratio of the total citations actually received by the denominator’s output, and the total citations that would be expected based on the average of the subject field. This way, an FWCI value equal to 1 means that the output performs just as expected for the global average. Values above 1 indicate that the output is cited more than expected according to the global average, while values below 1 indicate that the output is cited less than expected according to the global average. Taking into account the FWCI values, the documents in Table 2 present remarkable performance, with values above 3 in most cases; however, the document in the 5th position must be highlighted, since it attains a value above 11, which clearly indicates that this work has achieved a great impact.

Although further comments about the most important research trends will be introduced in the next sections after the analysis of the most employed author keywords and the bibliometric network analysis, the reading of the most cited publications gave an initial idea about some relevant issues that have attracted attention from researchers investigating selenium in water. According to this list of top cited papers, health and toxicological aspects of selenium have mainly captured the attention of the researchers along the analyzed period. The most cited article was a review that covered the hepatotoxicity and mechanism of action of haloalkanes [113]. It mentioned the antioxidant effects of selenium to mitigate the damage induced by these toxic compounds. Another article in the list (in the ninth position with 237 citations) investigated the role of selenium in alleviating the negative effects of aluminum [114], while other three articles were focused on the interactions between selenium and arsenic in the human body [115,116,117]. Moreover, the decreased risk of cancer and the preventive effects derived from diets containing adequate levels of selenium have been analyzed by two articles among the most cited [118,119]. Another relevant issue investigated by the other three papers in Table 6 is the identification of the presence of selenium and the quantification of the corresponding concentrations in different water, soil, dust, air and locally produced food samples, including the most important health risks and exposure levels [120,121,122]. Therefore, none of the 10 most cited documents cover aspects related to treatment alternatives to remove selenium and other metals from water, and, in fact, to find a document regarding this issue, the document in the 12th position in the ranking (216 citations) must be consulted, which explains the removal of some elements, including selenium, just by incorporation into hydrocalumite and ettringite [123]. This fact confirms again that the search of effective technical solutions to the problems caused by the presence of selenium in drinking water has not been a relevant research topic.

#### 3.1.5. Distribution Analysis of Author Keywords and Trending Topics of the Research

**Figure 3:**
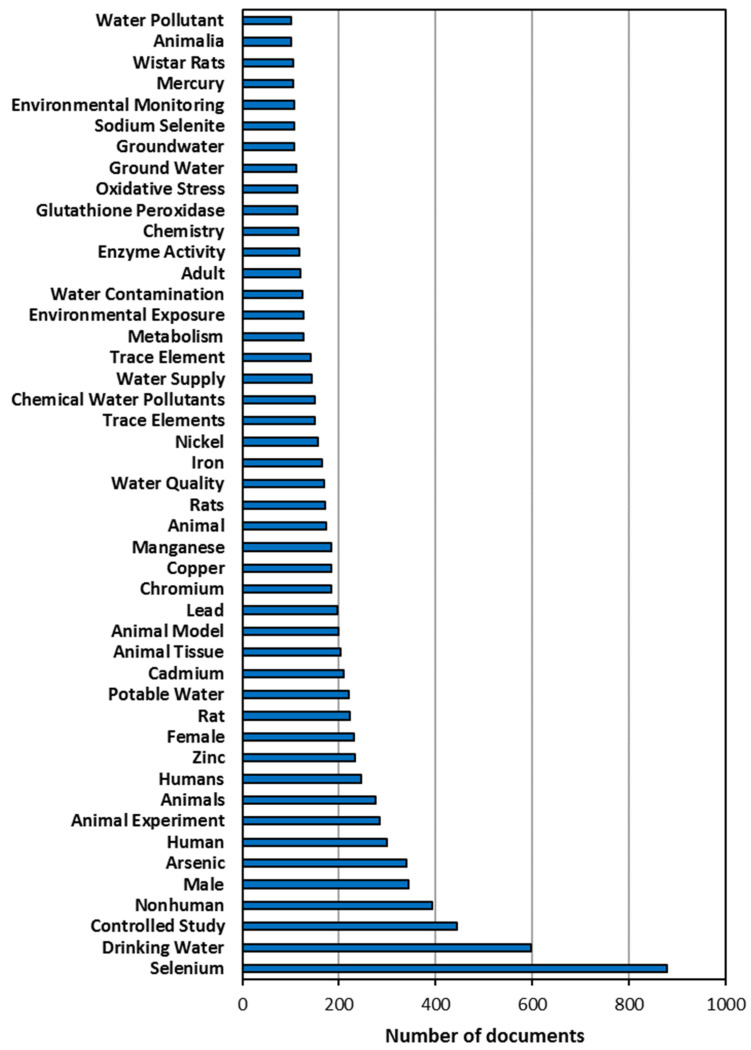
The top 46 most frequently used keywords.

The list of the 46 most often used keywords (the only ones that were mentioned at least 100 times) is shown in Figure 3. Obviously, it was clear that “*Selenium”* was the most frequent keyword as it was selected in 879 articles. The second positions of the ranking corresponded to the other expression selected to be introduced in the article title, abstract, keywords field of the search-engine database: “*Drinking water*” appeared 598 times. However, these figures indicated that only 69.6% of all the identified documents used “*Selenium*” as keyword, while the value for “*Drinking water*” decreased to 45.9%. Therefore, the selection of both expressions as keywords did not occur for more than half of the documents analyzed in this study. This fact pointed to the consideration of lower global concern and consequent scarcer research efforts about the presence of selenium in drinking water and the needs of treatment for its removal when compared to other metals or metalloids [124,125]. A further analysis of the keywords revealed a more important interest focused on the health and toxicological effects of selenium. The third position of “*Controlled study*” (444 times) in the keyword ranking underpinned this idea, confirmed also by the presence of other terms directly related to health studies, such as “*Nonhuman*”, “*Male*”, “*Human*”, “*Animal experiment*”, “*Female*” or “*Rats*” (all of them selected as keywords more than 220 times).

A further look at the results was enough to find other ten metallic and non-metallic elements in the ranking: “*arsenic*” (340 times), “*zinc*” (233 times), “*cadmium*” (209 times), “*lead*” (197 times), “*manganese*” (184 times), “*copper*” (184 times), “*chromium*” (184 times), “*iron*” (166 times), “*nickel*” (157 times) and “*mercury*” (105 times). On the one hand, the study of the synergistic and antagonistic effects selenium may cause on the toxicity of these other elements is a hot topic under investigation [12,126,127]. On the other hand, the evaluation of the presence and the distribution of these water pollutants implied general water sampling and characterization of the concentrations of all these elements in drinking water, water bodies or in different samples of environmental interest [61,128,129]. Lastly, among the top most frequent keywords, terms directly related to possible technologies for selenium removal in drinking water or wastewater treatments cannot be found.

#### 3.1.6. Bibliometric Network Analysis

In order to provide more information about of the most important research topics and their evolution through the studied period, a bibliometric network analysis based on science mapping was applied. This methodology is useful to analyze a field of research, since it allows the identification and visualization of the conceptual subdomains (particular themes or general thematic areas) and provides its thematic evolution throughout time [130]. Although various software tools are available for science mapping analysis, SciMAT software (Universidad de Granada, Granada, Spain) was selected due to its visual and easily understandable strategic diagrams and thematic evolution structure. SciMAT is an open-source science mapping software tool that can be freely downloaded, modified and redistributed according to the terms of the GPLv3 license [131].

The bibliometric network analysis conducted in this paper was based on four phases of analysis within a specified set of 4 periods (1990–2001, 2002–2011, 2012–2019 and 2020–2021). These periods were defined according to the different trends previously identified when the annual production was analyzed. The last period covers the years 2020 and 2021, which have suffered the pandemic lock-down. First, the research themes were identified using a frequency and network reduction of words (the value of the minimal frequency of a word to be considered was 3). The clustering algorithm used was the simple centers algorithm. To normalize data, Salton’s cosine was used to create the strategic diagram and the equivalence index was applied to normalize the co-word network of the thematic evolution structure. Secondly, the previously identified themes were then plotted on a bi-dimensional diagram composed of four quadrants, in which the vertical axis characterizes the density and the horizontal axis characterizes the centrality of a theme [132]. Thirdly, the results were organized in thematic network structures of themes as clusters, and the corresponding thematic evolution structure was obtained. These thematic network structures characterize the co-occurrence between the research themes and highlights the number of relationships and internal strength among them, while the thematic evolution structure provides an appropriate image of how the themes preserve a conceptual nexus throughout the defined subperiods. In both cases, the size of the clusters is proportional to the number of core documents and the links indicate cooccurrence among the clusters. Solid lines indicate that clusters share the main theme, and dashed lines represent the shared cluster elements that are not the name of the themes. The thickness of the lines is proportional to the inclusion index, which indicates that the themes have elements in common. Finally, the scientific contribution was measured by analyzing the most important research themes and thematic areas using the h-index, sum of citations, core documents centrality, density and nexus among themes [133].

**Figure 4:**
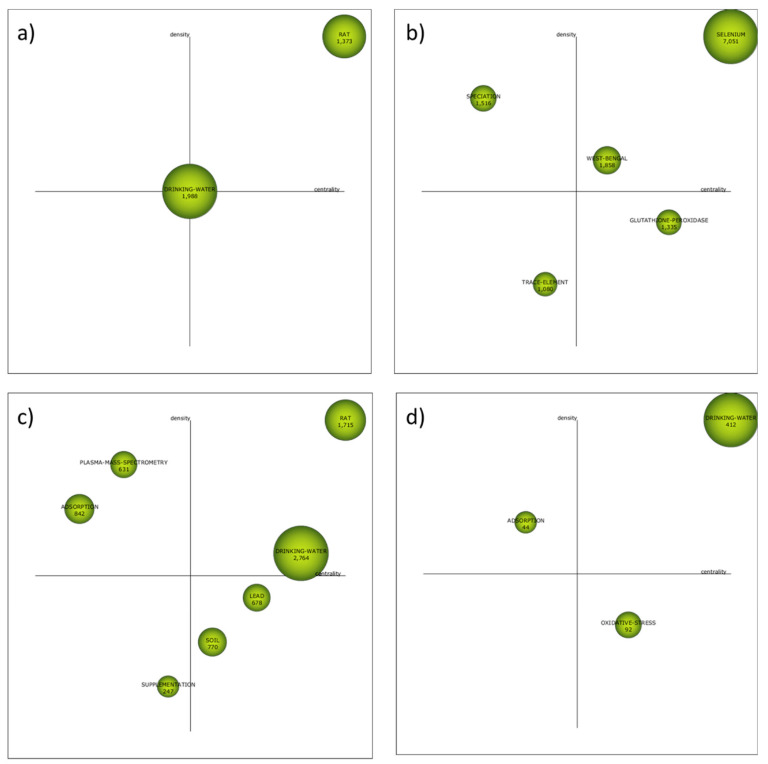
Strategic diagrams of the subperiods: 1990–2001 (**a**), 2002–2011 (**b**), 2012–2019 (**c**) and 2020–2021 (**d**).

The strategic diagram of each subperiod is depicted in Figure 4. According to their situation in these strategic diagrams, the themes can be classified into four different categories [134]:

– First quadrant (high centrality and high density): Motor themes.
  Trending themes for the field of research with high development.
– Second quadrant (high centrality and low density): Basic and transversal themes. Themes that are inclined to become motor themes in the future due to their high centrality.
– Third quadrant (low centrality and low density): Emerging or declining themes. Themes that require a more detailed analysis to define whether they are emerging or declining.
– Fourth quadrant (low centrality and high density): Highly developed and isolated themes. Themes that are no longer trending due to a new concept or technology.

The strategic diagrams present 17 clusters in total, 7 of them are motor themes, 4 are basic and transversal, 2 are emerging or declining themes and 4 are highly developed and isolated themes. The size of the clusters represents the number of total citations (the exact values appear in each cluster). In addition, h-index and absolute centrality and density values are presented for each cluster in Table 7.

**Figure 5:**
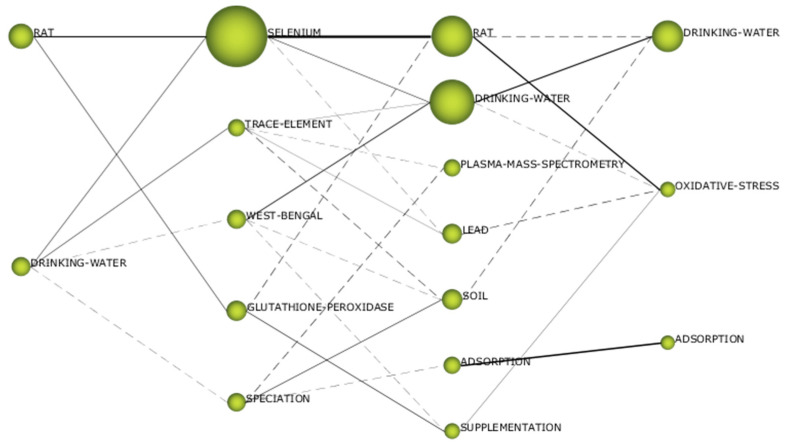
Thematic evolution structure of selenium and drinking water research (1990–2021).

**Figure 6:**
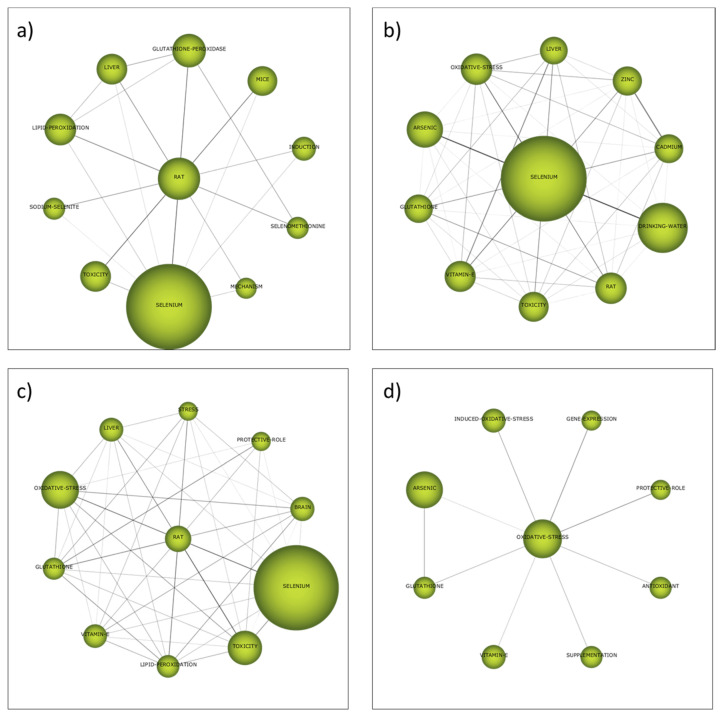
Evolution of the thematic network structure of the clusters *Rat/Selenium/Oxidative-stress*: 1990–2001 (**a**), 2002–2011 (**b**), 2012–2019 (**c**) and 2020–2021 (**d**).

**Figure 7:**
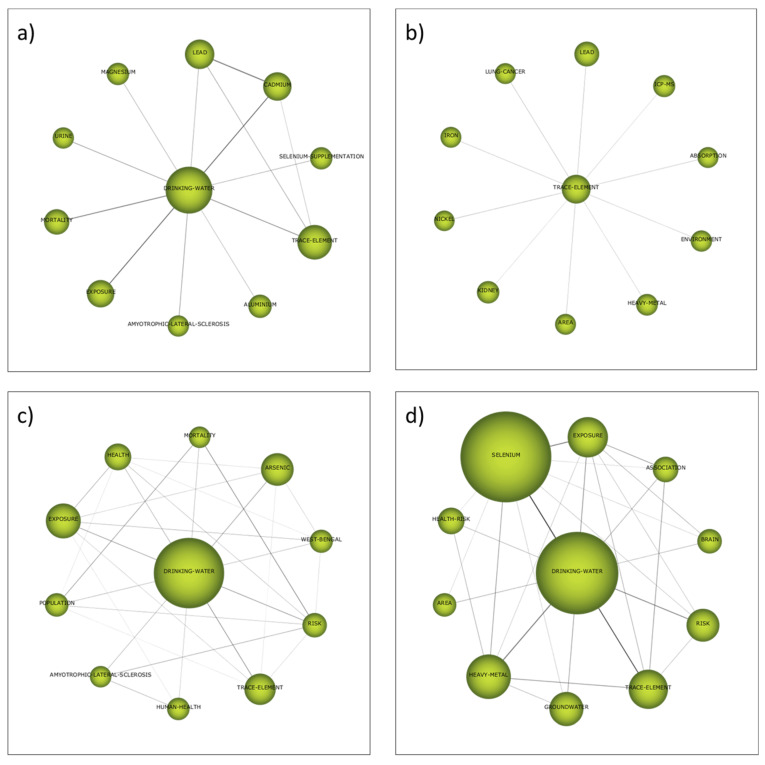
Evolution of the thematic network structure of the clusters *Drinking-water/Trace-element*: 1990–2001 (**a**), 2002–2011 (**b**), 2012–2019 (**c**) and 2020–2021 (**d**).

The thematic evolution structure is shown in Figure 5, which explains the evolution of the research field over the different subperiods considered in this study. In this way, each individual theme relevance is illustrated through its cluster size as well as with its relationships throughout the different subperiods. Two different continuity lines among clusters that cover all the time periods can be clearly identified. The first one includes the clusters *Rat*, *Selenium* and *Oxidative-stress*, while the second one is formed by the cluster *Drinking-water* and *Trace-element*. The thematic network structures of these two groups can be visualized in Figure 6 and Figure 7, respectively (while the network structures of the rest of clusters are compiled as Supplementary Material), which provide a good representation of the co-occurrence among keywords and allow the depiction of complex patterns.

The clusters *Rat* and *Selenium* were motor themes in the three first subperiods, but oxidative-stress was a basic and transversal theme in the last subperiod. The cluster selenium was the most cited one during the second subperiod (2002–2011), but the other clusters were less cited than the cluster *Drinking-water* in the corresponding subperiods. These clusters include the keyword “*Selenium*”, but in the case of the last subperiod, complemented with other keywords that give a direct link to health and toxicological aspects and animal testing, such as “*Toxicity*”, “*Liver*”, “*Rat*” or “*Mice*”. Among these topics, the role of selenium and the glutathione system in the context of defense against oxidative agents must be highlighted, since the keywords “*Oxidative-stress*”, “*Glutathione*”, “*Glutathione-Peroxidase*” and “*Antioxidant*” are included in these clusters. Therefore, the importance of the health and toxicological effects of selenium that was proposed from the analysis of the most frequent keywords is confirmed by the bibliometric network analysis.

The cluster *Drinking-water* (renamed *Trace-element* in the second subperiod) started as motor theme in the first subperiod, was an emerging theme in the second subperiod (2002–2011) and it returned to the first quadrant as motor theme in the last two subperiods. The list of keywords that belong to this cluster includes keywords such as “*Heavy-Metal*”, “*Lead*”, “*Cadmium*”, “*Arsenic*”, “*Iron*”, “*Nickel*” or “*Aluminium*”, which were more relevant in the first two subperiods. After the analysis of the most frequent keywords, two different reasons were presented to justify the presence of all these elements as keywords: the study of the synergistic and antagonistic effects selenium may cause on the toxicity of other elements and the evaluation of the presence and the distribution of these water pollutants in water sampling. The detailed study of the keywords included in the clusters identified in the bibliometric network analysis gave more relative importance to the first reason, the one related to synergistic and antagonistic toxicological effects, due to the presence of keywords such as “*Exposure*”, “*Health*”, “*Risk*”, “*Mortality*”, “*Lung*” or “*Kidney*”. In fact, keywords more directly related to the second reason, the one related to environmental sampling and measuring of these elements that are pollutants, can be found in other clusters with lower relevance. For instance, in the second subperiod, the cluster *Speciation* (Figure S3) included keywords such as “*Samples*”, “*Preconcentration*”, “*Water*”, ”*Soil*” and “*Atomic Absorption Spectrometry*”. This cluster was a highly developed and isolated theme, which gives an idea about the well-established technical solutions provided by analytical chemistry for speciation and quantification of selenium in environmental samples. Another cluster in the third subperiod, called “*Plasma-mass-spectrometry*”, also considered a highly developed and isolated theme, compiled more keywords regarding these analytical aspects, such as “*Water samples*”, “*Speciation Analysis*”, “*ICP-MS*”, “*HPLC-ICP-MS*”, “*Solid-Phase-Extraction*” and “*Atomic Absorption Spectrometry*” (Figure S4).

The lack of terms directly related to possible technologies for selenium removal from water during the analysis of the most frequently used keywords was at least partially solved when the cluster *Adsorption* was examined (Figures S7 and S9). This cluster appeared in the third subperiod (2012–2019) and had continuity until the last subperiod. In both cases it must be considered a highly developed and isolated theme, which was not trending. The list of keywords in this cluster included “*Removal*”, “*Arsenic-Removal*”, “*Sorption*”, “*Membrane*” and “*Selenate*”, which gave an idea about the most investigated technologies for selenium removal: the use of adsorbents and membrane-assisted separation processes.

The other cluster identified as motor theme not previously mentioned is *West-Bengal* during the second subperiod from 2002 to 2011 (Figure S1). This keyword in strongly correlated to Bangladesh and both regions suffered similar problems. On the one hand, uncontrolled industrial effluents are an important potential source of selenium pollution in these areas [135]. On the other hand, the dietary status of selenium is adversely affected by a chronic excessive ingestion of arsenic. These high levels of chronic arsenic ingestion from well water by people from these regions accelerate the excretion of selenium lowering the body’s content of this essential trace element [136]. Keywords such as “*Exposure*”, “*Contamination*”, “*Groundwater*”, “*Dietary-Selenium*” and “*Metabolism*”, which appeared in this cluster, confirmed this double problem that must be solved in these areas.

### 3.2. Review of Current Treatment Alternatives for Selenium Removal from Drinking Water

Although the health aspects of the presence of selenium in drinking water have been the focus of most research efforts covering this topic, the increasing interest from the scientific community in technical processes for removal of selenium from water has been proved by the publication of some recent reviews that cover this field [137,138,139,140,141,142,143]. The list of commercially available and emerging technological options for selenium removal is extensive, but all the alternatives can be included in one of the following main categories:

- Adsorption and ion exchange.
- Coagulation-flocculation-precipitation.
- Membrane-based processes.
- Biological treatments.

Since more detailed reviews are available, the aim of this section is just to mention the most important trending topics identified as a consequence of the bibliometric analysis, without the intention of compiling a concise register of all the scientific bibliography published about technical solutions to remove selenium from water.

#### 3.2.1. Adsorption and Ion Exchange

This treatment category can be considered the most important one according to the number of papers published and the relevance of them. In fact, the unique article included among the most cited ones in Table 6 that presented results of a technical solution for selenium removal from water was related to the use of anionic clay minerals based on aluminum hydroxides as adsorbents/ion exchangers [123].

Iron compounds have certainly been the most recurrent materials tested as adsorbents for selenium removal from water. Iron oxides, hydroxides and oxyhydroxides, as well as zero-valent iron (ZVI), have frequently been reported as efficient adsorbents for selenium oxyanions [144,145,146,147,148,149,150,151,152,153,154]. The mechanisms that rule selenium oxyanions adsorption on iron compounds have been determined and modelled [155] and the effects that pH, surface loading, and ionic strength have on these mechanisms have been reported [156]. Selenite was more effectively removed that selenate by natural iron oxides (goethite and hematite) under identical conditions [157] and equivalent results were confirmed for other iron compounds derived from corrosion of ZVI [158]. Iron compounds resulted in a cost-effective solution, since they are not expensive and some are even waste materials, such as water treatment residuals, bauxite-processing red mud or fly ashes, can be directly reused as sorbents [159,160]. An innovative approach pointed to the employment of nanoparticles, nanocomposites and other nanomaterials for the intensification of selenium removal [161,162]. ZVI must be considered a very appropriate option for selenium removal, since it is highly reactive and widely available. ZVI can be easily oxidized by dissolved oxygen, contaminants themselves or even just water, resulting in iron oxides, hydroxides and oxyhydroxides as aqueous corrosion products [163]. Enhancing the corrosion of ZVI has been observed as an effective approach to promote its decontamination performance and the role of additional oxidants in this promotion has gained relevance. The addition of chemicals, such as hydrogen peroxide, sodium hypochlorite or potassium permanganate, achieved highly efficient and rapid selenium oxyanion removal [164,165,166]. Moreover, some treatment proposals have taken advantage of the magnetic properties of some iron-based adsorbents to improve the performance of the process by application of magnetic fields [167,168,169].

The removal of selenium from water by other metallic compounds, specifically oxides and hydroxides, has been reported. Activated alumina adsorption is known to be an effective and inexpensive technology for the removal of metals from drinking water and has been successfully applied to the case of selenium oxyanions [170,171,172,173,174,175]. Once again, activated alumina was more effective for selenite adsorption than selenate [176,177]. Other research works have proposed the employment of metallic oxides, such as titania, silica or zirconia, for this same purpose [178,179,180,181,182]. In some cases, the developed adsorbents were highly selective to selenite, even in the presence of selenate or selenide [183,184]. Natural and modified zeolites are high-performance adsorbents that have been implemented in the treatment of drinking water and have demonstrated that the selenium limits for drinking water can be achieved with specific process designs based on these aluminosilicates [185,186,187].

The removal of toxic oxyanions from water by means of adsorption onto carbon is a well-known process and an increasing number of drinking water treatment plants have installed activated carbon filters as secondary or tertiary treatments for the removal of micropollutants [188]. Both granular activated carbons (GACs) and powdered activated carbons (PACs) have been applied to the removal of selenium and the results revealed practical total removal of selenite (initial 100 µg/L concentration) with contact times not longer than 60 min under acidic or neutral conditions, but worse performance under alkaline pH [189]. However, the results with higher initial concentrations (5–75 mg/L solutions) demonstrated that, although relatively significant removal (87%) was observed for the lowest concentration tested, higher concentrations resulted in reduced removal percentages [171]. Besides, the use of activated carbons as supports to form stable composites loaded with metallic compounds has been reported. This way, the physical sorption that characterized the retention of selenium oxyanions in activated coals can be completed with the chemical adsorption provided by metal oxides and hydroxides, such as iron or copper [190,191]. This combination enhanced the removal of selenium oxyanions, particularly for selenate, which was only partially removed by activated carbons [192].

Layered double hydroxides (LDHs), also called anionic clays, contain positive-charged layers and counter-anions in the interlayer space. They are ordered according to the generic layer sequence [OHM^2^OH A OHM^3^OH]_n_, where M^2^ and M^3^ represents layers of divalent and trivalent metal cations, respectively, OH are layers of hydroxide anions, and A are layers of counter-anions. These materials have demonstrated effective removal of oxyanions from water due to the combination of adsorption in their large surface area and high anion exchange capacity. For the case of selenium removal, Al^+3^ was clearly the most preferred trivalent cation of the LDHs evaluated [193,194,195], but examples of Fe^+3^ in combination with Zn^+2^ can be found [196,197]. The tests with Mg/Al and Zn/Al LDHs with intercalated chloride revealed that the oxidation state of selenium was not too relevant, since the adsorption trends for both selenite and selenate on these LDHs were similar under the experimental conditions [198]. The presence of zwitterions instead of the classical anions in the interlayer space can imply a better adsorption capacity and selectivity for the removal of oxyanions. As example, the use of the amino acid glycine replacing nitrate in a Ni/Al LDHs increased the removal of selenate from 34 to 83% [199]. Another innovative approach identified was the loading of ZVI in a Mg/Al LDH, which enhanced the removal of selenate by incorporation of reductive immobilization mechanisms [200]. Nevertheless, the removal of selenium by LDHs can be severely affected and even inhibited by the presence of competitive anions in the water to be treated [201].

The application of commercial ion exchange resins for selenium removal has been reported. Articles describing the performance of strong and weak basic anionic resins in the presence of selenite and selenate are common [202,203,204,205,206,207], although these ion exchange processes have to deal with two important disadvantages. On the one hand, the occurrence of other oxyanions, such as nitrate or sulfate, implies a strong competition for the sorption sites in the resins. Since typically the concentrations of these competitive anions are several orders of magnitude higher that the selenium concentrations, resin can be exhausted rapidly and selenite and selenate removal inhibited [208]. On the other hand, ion exchange resins do not appear to be the most economical option, especially when compared to alternative adsorbents, which result in being clearly cheaper [197]. Consequently, research efforts have been focused on the search for solutions to improve these two drawbacks that ion exchange resins present. Low-cost ion exchangers derived from waste biomass [209,210] and inorganic ion exchangers based on silicates [211] have been investigated for selenium removal with successful results and can be considered a valid option to reduce the economic costs of the process. In order to improve the selectivity of the resins for selenium oxyanions and avoid the competition of other anions, innovative ligand and chelating resins [212,213,214] and metal-loaded cationic resins [215] have been proposed. Nevertheless, further research efforts are still required to identify more selective ion exchangers for the removal of selenium from aqueous solutions.

#### 3.2.2. Coagulation-Flocculation-Precipitation Followed by Filtration

The direct precipitation of selenium compounds is not an adequate technology for selenium removal from water. Selenite and selenate oxyanions are the most frequent species of selenium in waters and, in contrast to the low solubility of metallic selenides, most metallic selenites and selenates are soluble in water [3,216]. However, the precipitation of Se^+4^ by sulfide ions is a well-known process, although the nature and characteristics of the solids formed in selenium-sulfide systems are not totally defined. The role of selenium disulfide (SeS_2_) is crucial, but sulfur and selenium are miscible in all proportions and can form complex polymer-like molecules, thus the sulfur-selenium solid solutions are composed of cyclic Se–S rings containing a variable number of Se and S atoms [217]. Selenite removal at neutral pH by reductive precipitation using sodium sulfide as reducing agent (with S/Se molar ratios between 1.5 and 11) has been investigated and the precipitation reaction went to completion with less than 5 µg/L of soluble selenium remaining in solution after 10 min at ambient temperature [218,219].

Ferric coagulants, such as FeCl_3_ or Fe_2_(SO_4_)_3_, are frequently used in water treatments due to their availability and low price. Some metals and metalloids species can co-precipitate or adsorb onto the surface of these ferric coagulants. Selenite behaves in this way and is readily removed through ferric co-precipitation. However, this treatment method is not adequate for selenate [220], but a previous reduction pretreatment, for example with sulfite, has been successfully applied to transform selenate into selenite and remove it by ferric coagulation [221]. Once again, selenium concentration below 5 µg/L in the treated effluent were achieved by ferric coagulants [222]. Aluminum coagulants, such as AlCl_3_ or poly-aluminum chloride PAC, are very commonly used for water treatment too, since Al cations hydrolyze quickly and form abundant hydroxide precipitates in situ, which can act in a similar way to their homologous ferric compounds. Nevertheless, the research about the use of aluminum coagulants for selenium removal has demonstrated that the use of ferric compounds was preferred, since they were much more efficient [223,224]. Nevertheless, the performance of the coagulation process can be enhanced with the addition of commercially available polymeric flocculants, which enhanced the removal of selenium [225].

In electrocoagulation, an electrical current is used to generate metallic ions from a sacrificial anode immersed in the water to be treated. This way, continuous in situ generation of ions that polymerize rapidly and act as coagulants is possible. Although aluminum sacrificial anodes have been tested with satisfactory results [226], iron has been most frequently selected due to better sedimentability properties of the precipitated particles [227]. Regardless of the applied coagulation and flocculation process, filtration is required to remove the particles and microfiltration membranes [228] or alternative filtration media, such as sand filters [229], must be implemented as post-treatment.

#### 3.2.3. Membrane-Based Processes

The removal of toxic metals and metalloids from environmental aqueous samples with high salinity has been a rising area for membrane separation, because, under these circumstances, they provide a better solution than the technologies explained in the two previous sections, which can suffer worse performance due to elevated ionic contents. Pressure assisted membranes are good candidates for the removal of selenium, but the selection of the most appropriate technology must take into account the balance between high permeate production and efficient selenium rejection.

One the one hand, the strictest membranes, such as reverse osmosis (RO) and tight nanofiltration (NF), produce relatively low permeation fluxes and require high applied pressure, but the rejection percentages are maximal. On the other hand, less restrictive membranes, such as loose NF or ultrafiltration (UF), are characterized by production of larger volumes of permeate, but the removal performances are often considerably lower [230].

Although RO and NF are the most frequent pressure-assisted membrane technologies selected to remove selenium from water because of the small size of the selenium oxyanions, which is around 2.4 Å [137], an example of the application of UF to eliminate selenium was found [231]. This work investigated the potential to remove both Se^+4^ and Se^+6^ states by different polymeric and ceramic membranes. On the one hand, the use of commercial polyamide UF membranes with MWCO values between 2.5 and 3.5 kDa implied high permeate fluxes (more than 5 × 10^−5^ m^3^/m^2^·s) and rejection percentages around 90% and 95% for Se^+4^ and Se^+6^, respectively, with very little influence of the initial selenium concentration. The higher rejection of Se^+6^ can be justified by its stronger electrostatic interactions, since the charge of Se^+6^ oxyanions was higher than the one corresponding to Se^+4^ oxyanions for most pH values. The most extreme case was the rejection at pH 1.5, where neutral H_2_SeO_3_ is not rejected by the UF membrane while HSeO_4_^−^ showed a rejection value above 40% (similar case when HseO_3_^−^ was compared to SeO_4_^−2^). Even when the charge of the oxyanions were equal, steric effects favored the rejection of Se^+6^ versus Se^+4^. The importance of the electrostatic interaction in the rejection of selenium oxyanions was confirmed by the decreased rejection values due to higher ionic strength. On the other hand, the performance of a ceramic UF membrane with MWCO 8 kDa exhibited lower rejection percentages: around 30% and 80% for Se^+4^ and Se^+6^, respectively (although the permeate flux doubled the values of the polymeric membranes). However, these rejection values were greatly improved by addition of chitosan as chelating agent to improve selenium removal.

The first examples of the application of RO to selenium removal were published in the late 1970s and 1980s [140]. In contrast to arsenic, where the oxidation state of the element highly determined the performance of the membrane process due to the presence of neutral species of As^+3^ [93], selenium appears as negatively charged oxyanions in most environmental water samples and both Se^+4^ and Se^+6^ are efficiently rejected by RO membranes [232]. Several studies have analyzed in detail the permeability of selenium species through RO membranes and the interactions with other ions in aqueous solution [233,234]. Nevertheless, RO can be an inadequate solution to treat streams with excessive salinity, due to the extreme osmotic pressure these types of solutions present. As an example, deep formation water, which is extracted as an undesired byproduct from oil production wells, can be mentioned, since its hypersalinity requires pressure conditions exceeding 200 bars across the RO membrane [235]. Some illustrative case studies of the application of RO to the removal of selenium are compiled in Table 8. The rejection percentages attained by RO membranes are higher than 94%, with some examples around 100%, but the lower rejection values corresponded to initial selenium concentrations below 100 µg/L. However, the permeate fluxes are significantly reduced and due to the balance between simultaneous high water permeability and rejection that NF presents, it can be considered a most adequate technology to achieve this goal [236]. Table 9 introduces some relevant articles that covered the treatment by NF of water samples with high selenium content. An analysis of the results of these works pointed to a more valuable compromise solution by implementation of NF. The rejection of selenium maintained equivalent values to those obtained by RO, but NF provided increased permeate production, with values at least an order of magnitude higher than the case of RO [237].

Furthermore, apart from pressure-assisted membrane technologies, other innovative membrane technologies have been investigated for selenium removal. Firstly, supported liquid membranes, which have been successfully applied to the removal of other metals and metalloids from water, have been investigated for the case of selenium. Three different stages and phases are involved in supported liquid membranes: solute extraction from the feed phase, diffusion of solute through the extractant-containing phase and stripping of solute to the acceptor phase. For the particular case of selenium, several investigations have been completed, all based on the use of feed and acceptor aqueous phases, while the extractant is an organic phase. Mafu et al. employed Aliquat 336 supported on PP (polypropylene) hollow fibers to transfer selenium to a 0.8 M NaOH stripping solution [248]. From an initial 100 µg/L concentration, the selenium content was reduced by 78%. Lower removal percentages (around 60%) were achieved by Ambe et al., which selected TBP (tributyl phosphate) as carrier in a decalin phase supported on microporous PTFE (polytetrafluoroethylene) disks [249]. In this case, HCl solution was employed as acceptor phase. Selective removal of selenium compared to other metallic impurities in aqueous solution was demonstrated by Noguerol et al., in this case with NaDDTC (sodium diethyldithiocarbamate) as carrier in kerosene phase supported on PTFE (polytetrafluoroethylene) membrane and with H_2_O_2_ as stripping agent [250]. Secondly, brackish groundwater was treated by pervaporation to be used for micro-irrigation [251,252]. In pervaporation, the membrane acts as a selective barrier between the two phases: the liquid-phase feed and the vapor-phase permeate. It allows the desired components of the liquid feed to transfer through it by vaporization, and consequently, the separation of the components is based on a difference in transport rate of individual components through the membrane. Among the model compounds selected for these pervaporation studies, Se^+6^ was included. Under optimal configuration of the sweeping gas pervaporation system, the maximal permeate water flux was 5.1 × 10^−8^ m^3^/m^2^·s. The removal of selenium from solutions with initial concentrations in the range 56–154 µg/L attained 92% with corrugated sheet membranes made of thermoplastic copolyether esters elastomers. Lastly, electrodialysis was applied to the removal of inorganic trace contaminants (including selenium) from a real brackish groundwater in a remote Australian community [253]. Electrodialysis is based on the transport of salt ions from one solution through ion-exchange membranes to another solution under the influence of an applied electric potential difference. A systematic investigation of the most relevant operation conditions (applied voltage and solution pH) was completed to elucidate removal efficiency. A higher applied voltage enhanced removal of Se^+6^ (from 33 to 48%) at pH 7, but the adjustment of the pH value was a more effective measure to improve the removal. On the one hand, pH below 6 increased the removal percentage above 80%, while, on the other hand, pH values between 8 and 11 formed insoluble CaSeO_4_, which eliminated selenium from the water but caused fouling of the membrane.

#### 3.2.4. Biological Treatments

**Figure 8:**
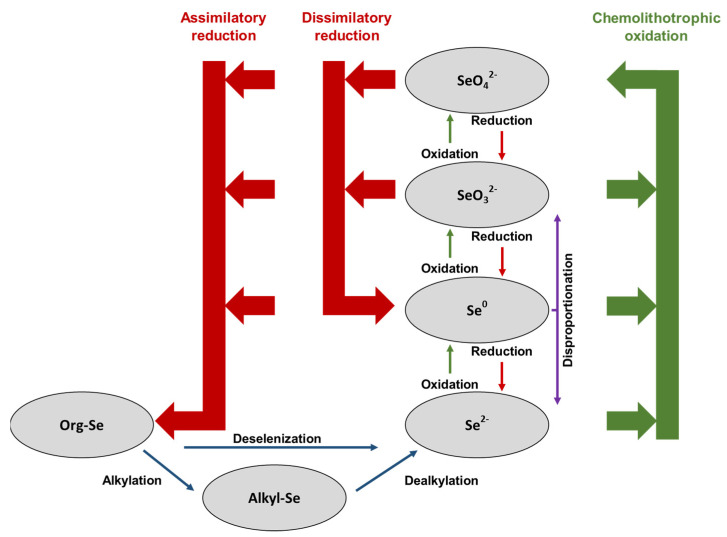
Selenium transformations in nature.

The reactions that are involved in the biogeochemical cycle of selenium have been deeply investigated, including the ones more directly related to microorganisms, which are depicted in Figure 8 [254]. Among all these transformation reactions, dissimilatory reduction pathways must be considered the most interesting option in terms of biological removal of selenium from water [255,256,257,258]. Many microorganisms that transform the soluble selenate and selenite oxyanions into insoluble elemental selenium have been identified and isolated from pristine [259,260,261] and polluted environments [262,263,264,265] for better understanding of the metabolic mechanisms involved [143]. A compilation of major cultured selenium-reducing microorganisms and their main properties has been published [4].

Biological selenium removal by environmentally sustainable technologies is an attractive alternative due to the water characteristics (dilute selenium concentration and high volume to be treated) and low costs. In addition, adequate biological treatment may imply the transformation of dissolved selenium into a recoverable insoluble form. The recovery of the elemental red selenium resulting from dissimilatory reduction is seriously considered to reduce or even compensate the treatment costs. Although several studies have demonstrated that these bacterial selenium nanoparticles can contain impurities, such as heavy metals or organic compounds, the recovery of selenium provides high value for the industrial sectors interested in its applications [266]. Nevertheless, the recovery of the biogenic elemental selenium is challenging, since it exhibits colloidal properties that require further post-treatment (filtration, centrifugation, coagulation, electrocoagulation, etc.) for separation of the colloidal selenium from the treated water [226,267].

The dissimilatory reduction of selenate and selenite has been investigated under different scenarios and it was successfully applied under methanogenic, sulfate reducing, denitrifying or hydrogenotrophic conditions [268]. All these tests have demonstrated that the dissimilatory reduction to elemental selenium is viable even in presence of high concentrations of other oxyanions, such as sulfate or nitrate [263,269,270,271]. In addition, the presence of heavy metals did not exert a significant effect on selenite microbial reduction [272]. The biological treatment of selenium-polluted water required the enrichment and retention of microorganisms in bioreactors. Different bioreactor configurations have been analyzed for this purpose. Among all the alternatives tested, which include from basic anaerobic ponds to complex bio-electrochemical systems [273,274,275], fluidized bed reactors (FBRs), upflow anaerobic sludge blankets (UASBs) and membrane biofilms reactors (MBfRs) must be highlighted [276].

In FBRs, a biofilm is formed on added solid particles, which are fluidized by the movement of the liquid to be treated, avoiding the transport limitations that appear in stationary-bed processes. For the removal of selenium, examples of activated carbon and commercial supports, such as Kaldness-K1 or Extendospheres, as added solid particles have been investigated [277,278,279]. Optimization of the bioreactor operation conditions resulted in selenium removal percentages above 88% with hydraulic retention times no longer that 0.5 h. Meanwhile, UASBs employ beds of granular sludge developed by the self-aggregation of microorganisms, which are fed from the bottom with water to be treated, while a gas-liquid-solid separator in the upper section of the bioreactor retains the biomass. Different types of granular sludge have been investigated for selenium microbial reduction, with removal values above 90%, even when the selenium concentration in the influent exceeded 3 mg/L [280,281]. Finally, a type of MBfR has been systematically studied in the last decade for the removal of selenium oxyanions: the H_2_-based MBfR [282]. These bioreactors consume non-toxic gaseous hydrogen as electron donor for the reduction of dissolved pollutants. The gas is delivered by diffusion through the walls of non-porous hollow fiber membranes and a biofilm is naturally formed on the outer wall of the membranes [283]. Different interactions between selenium oxyanions and other anions present in the influent, such as nitrate or sulfate, have great influence on the performance of MBfRs due to the direct link between the specific microbial community structure in the biofilm and the composition of the aqueous solution medium [284,285,286]. Once again, the decrease of the selenium content in the water treated in these bioreactors ranged between 90 and 99%, even with initial concentration from 1 to 11 mg/L, values more than one order of magnitude higher than the maximum contaminant level for drinking water [287,288,289].

Phytoremediation takes advantage of the ability of some plants and their related microbes to take selenium from the environment [290,291]. The design of constructed wetlands is a valuable green option to apply the potential of these plants for improving water quality. The main mechanisms for removal of selenium in constructed wetlands include biosorption, biologically-mediated precipitation, assimilation and accumulation, and volatilization of organic selenium compounds produced via bioalkylation [292]. The relative contribution of each pathway and the precise roles of the plants, the corresponding microorganisms and even the animals that may be present depends on the specific biotic community and the abiotic conditions in the constructed wetland. The identification of the most adequate plant species for selenium removal is a key aspect of the research in this field. Cattails (*Typha* spp.) have demonstrated a satisfactory performance to effectively reduce the selenium concentration in waters, but other species, such as bulrushes (*Cyperus* spp., *Scirpus* spp., *Schoenoplectus* spp.), reeds (*Phragmites* spp.), saltgrass (*Distichlis* spp.), rabbitfoot grasses (*Polypogon* spp.) or trees, such as poplars (*Populus* spp.), must be mentioned too as adequate candidates for selenium removal [293,294,295].

In addition, microalgae, such as *Chlorella vulgaris*, have been successfully applied to the removal of selenium [296,297]. The percentage of removal of selenium was highly dependent on the exact conditions of each constructed wetland, but the most frequent values ranged from around 40–50% as minimal values [298,299] to practically complete removal in the most effective cases [297,300,301], with many case studies around 75% removal [302,303,304,305,306].

Biological methods must be considered efficient ways to reduce the selenium concentration in water. These methods allow the recovery of selenium as insoluble forms or selenium-enriched vegetables can be produced, which exhibit interesting antioxidant properties [299]. Therefore, research efforts must be promoted in this field in order to demonstrate the technical and economic viability of real scale processes and pave the way to further implementation.

## 4. Conclusions

A summary of the research on selenium in drinking water was prepared from the results of a bibliometric analysis (information about annual publications, document types, languages, countries, institutions, categories, journals and keywords). The number of accumulated publications about this subject increased according to a quadratic evolution during the 1990–2019 period. The USA was the leading country in total number of publications, followed by a couple of Asian countries (China and India). In fact, Chinese institutions appeared among the most productive ones. Although *Environmental Science* was the most frequent category, many studies in *Medicine* and *Biochemistry, Genetics and Molecular Biology* have investigated the identification of the health effects of the intake of selenium. In fact, these aspects directly related to the consequences of selenium intake on the human health have been identified as the most deeply investigated. The bibliometric network analysis revealed that the clusters with keywords in this field were more relevant and they were cited a higher number of times than the clusters with keywords more easily related to water treatment.

Although the search of effective technical solutions to solve the problems caused by the presence of selenium in drinking water has been less intensive than the treatments of other pollutants, such as arsenic, many research works have investigated the best practices to remove selenium oxyanions. Adsorption was by far the most investigated treatment alternative. Several metallic compounds, mainly iron and aluminum oxides, hydroxides and oxyhydroxides, are the most relevant sorbents under study. Nevertheless, further research efforts to identify more selective ion exchangers must be recommended. Pressure-assisted membrane technologies (mainly nanofiltration and reverse osmosis) must be considered competitive solutions, but a balance between selenium rejection and permeate production is required. Processes for selenium removal based on coagulation, flocculation and precipitation have not gained too much attention by researchers, although iron and aluminum salts have been successfully employed as coagulants, especially for the retention of colloidal elemental selenium particles. The most relevant biological treatments take advantage of the dissimilatory reduction of selenate and selenite to elemental selenium. Moreover, the recovery of this elemental selenium could be a sustainable option to close the cycle of selenium, thus the investigation related to the biological methods that can close this loop must be promoted, including the production of selenium-enriched vegetables.

## Figures and Tables

**Table 1:** The languages employed by the publications.

Language	Publications	Contribution (%)
English	1050	94.0
Chinese	33	3.0
Russian	13	1.2
French	7	0.6
Czech	3	0.3
Japanese	3	0.3
Spanish	3	0.3
Bulgarian	2	0.2
German	2	0.2
Hungarian	2	0.2
Moldavian	2	0.2
Romanian	1	0.1
Ukrainian	1	0.1

**Table 2:** The top 31 most productive countries (at least 10 documents).

Country	Publications	Contribution (%)	Publications/ Million Habitants	Publications/ Trillion US Dollars GPD
United States	259	23.2	0.786	12.4
China	172	15.4	0.122	11.8
India	104	9.3	0.075	41.6
Canada	61	5.5	1.605	37.9
Japan	46	4.1	0.366	10.5
Turkey	44	3.9	0.522	43.1
Italy	37	3.3	0.621	21.3
Germany	36	3.2	0.432	10.5
United Kingdom	36	3.2	0.536	12.5
France	34	3.0	0.505	14.1
Russian Federation	33	3.0	0.229	23.2
Brazil	32	2.9	0.151	18.3
Sweden	31	2.8	2.995	58.2
Spain	30	2.7	0.634	25.4
Egypt	29	2.6	0.283	70.4
Tunisia	27	2.4	2.285	613.6
Saudi Arabia	25	2.2	0.718	38.4
Bangladesh	24	2.1	0.146	88.6
Pakistan	21	1.9	0.095	65.7
Iran	20	1.8	0.238	48.8
Poland	20	1.8	0.527	36.0
Nigeria	18	1.6	0.087	36.4
Australia	17	1.5	0.662	11.4
Norway	14	1.3	2.602	34.7
Austria	12	1.1	1.348	31.1
Belgium	12	1.1	1.038	25.7
Denmark	12	1.1	2.058	36.6
South Africa	12	1.1	0.202	35.7
Switzerland	12	1.1	1.390	16.2
Greece	11	1.0	1.027	59.5
Czech Republic	10	0.9	0.935	49.3

**Table 3:** The top 18 most productive institutions (at least 10 documents).

Institution	Publications	Contribution (%)
Chinese Academy of Sciences (CHINA)	40	3.6
Institute of Geographical Sciences and Natural Resources Research (CHINA)	19	1.7
University of Sfax (TUNISIA)	16	1.4
Università degli Studi di Modena e Reggio Emilia (ITALY)	15	1.3
University of Chinese Academy of Sciences (CHINA)	15	1.3
Panjab University (INDIA)	14	1.3
Environmental Protection Agency (USA)	14	1.3
Ministry of Education (CHINA)	13	1.2
Northeast Agricultural University (CHINA)	13	1.2
The University of Chicago (USA)	13	1.2
CHU Habib Bourguiba (TUNISIA)	13	1.2
University of Calgary (CANADA)	12	1.1
Karolinska Institutet (SWEDEN)	12	1.1
University of Saskatchewan (CANADA)	12	1.1
Columbia University (USA)	12	1.1
Universidade de São Paulo (BRAZIL)	10	0.9
Columbia Mailman School of Public Health (USA)	10	0.9
Universidade Federal de Santa Maria (BRAZIL)	10	0.9

**Table 4:** The top 9 most popular subject categories (at least 30 documents).

Ranking	Subject	Publications	Contribution (%)
1	Environmental Science	471	42.2
2	Medicine	359	32.1
3	Biochemistry, Genetics and Molecular Biology	281	25.2
4	Chemistry	226	20.2
5	Pharmacology, Toxicology and Pharmaceutics	164	14.7
6	Agricultural and Biological Sciences	105	9.4
7	Earth and Planetary Sciences	59	5.3
8	Engineering	49	4.4
9	Chemical Engineering	47	4.2

**Table 5:** The top 8 most popular journals (at least 15 documents).

Source	SJR 2020 (Scopus)	Publications	Contribution (%)
Biological Trace Element Research	0.649	54	4.8
Science of the Total Environment	1.795	44	3.9
Environmental Science and Pollution Research	0.845	19	1.7
Environmental Research	1.460	18	1.6
Environmental Science and Technology	2.851	18	1.6
Environmental Health Perspectives	2.257	16	1.4
Environmental Monitoring and Assessment	0.590	15	1.3
International Journal of Environmental Research and Public Health	0.747	15	1.3
Journal of Trace Elements in Medicine and Biology	0.739	15	1.3

**Table 6:** The top 10 most cited papers.

Ranking	Articles	Times Cited	FWCI
1	Title: **Hepatotoxicity and mechanism of action of haloalkanes: Carbon tetrachloride as a toxicological model** Authors: Weber, L.W.D., Boll, M., Stampfl, A. Source: *Critical Reviews in Toxicology* Published: 2003	1278	3.37
2	Title: **Metals and micronutrients—Food safety issues** Authors: McLaughlin, M.J., Parker, D.R., Clarke, J.M. Source: *Field Crops Research* Published: 1999	729	4.97
3	Title: **Lung cancer in never smokers: Clinical epidemiology and environmental risk factors** Authors: Samet, J.M., Avila-Tang, E., Boffetta, P., (...), Thun, M.J., Rudin, C.M. Source: *Clinical Cancer Research* Published: 2009	348	3.73
4	Title: **Arsenic exposure and cardiovascular disease: A systematic review of the epidemiologic evidence** Authors: Navas-Acien, A., Sharrett, A.R., Silbergeld, E.K., (...), Burke, T.A., Guallar, E. Source: *American Journal of Epidemiology* Published: 2005	300	3.18
5	Title: **The effects of arsenic exposure on neurological and cognitive dysfunction in human and rodent studies: A review** Authors: Tyler, C.R., Allan, A.M. Source: *Current Environmental Health Reports* Published: 2014	274	11.79
6	Title: **Survey of arsenic and other heavy metals in food composites and drinking water and estimation of dietary intake by the villagers from an arsenic-affected area of West Bengal, India** Authors: Roychowdhury, T., Tokunaga, H., Ando, M. Source: *Science of the Total Environment* Published: 2003	251	6.13
7	Title: **Trace elements and cancer risk: A review of the epidemiologic evidence** Authors: Silvera, S.A.N., Rohan, T.E. Source: *Cancer Causes and Control* Published: 2007	248	3.10
8	Title: **Health risks from the exposure of children to As, Se, Pb and other heavy metals near the largest coking plant in China** Authors: Cao, S., Duan, X., Zhao, X., (...), He, B., Wei, F. Source: *Science of the Total Environment* Published: 2014	242	7.48
9	Title: **Antioxidant effect of vitamin E and selenium on lipid peroxidation, enzyme activities and biochemical parameters in rats exposed to aluminium** Authors: El-Demerdash, F.M. Source: *Journal of Trace Elements in Medicine and Biology* Published: 2004	237	2.08
10	Title: **Strategies for safe and effective therapeutic measures for chronic arsenic and lead poisoning** Authors: Kalia, K., Flora, S.J.S. Source: *Journal of Occupational Health* Published: 2005	226	3.72

**Table 7:** Citations, h-indexes and centrality and density values of the different clusters identified in the bibliometric network analysis.

Cluster	Citations	h-Index	Centrality	Density
Subperiod 1 (1990–2001)				
Rat	1373	27	49.60	19.06
Drinking-water	1988	16	19.10	13.82
Subperiod 2 (2002–2011)				
Selenium	7051	49	125.8	33.01
West Bengal	1858	35	28.34	17.44
Glutathione-peroxidase	1335	31	54.04	11.51
Speciation	1516	30	16.69	17.80
Trace-element	1080	29	26.05	4.03
Subperiod 3 (2012–2019)				
Rat	1715	30	66.49	34.81
Drinking-water	2764	30	59.20	15.56
Soil	770	26	26.81	10.33
Lead	678	23	30.13	11.80
Supplementation	247	23	17.17	3.23
Plasma-Mass-Spectrometry	631	19	16.83	18.18
Adsorption	842	17	5.14	17.56
Subperiod 4 (2020–2021)				
Drinking-water	412	8	63.42	25.93
Oxidative-stress	92	7	20.11	8.97
Adsorption	44	4	18.96	19.33

**Table 8:** Examples of application of reverse osmosis for selenium removal from water.

Treated Water	Membrane	ΔP (bar)	Permeate Flux (m^3^/m^2^·s)	Initial [Se] (µg/L)	Removal (%)	Reference
Agricultural drainage water	-	55	1.1 × 10^−7^	30,000	99.9	[232]
Mining polluted groundwater	PAC1/TW30 (Ionics/Filmtec)	7	-	550	98	[230]
Synthetic aqueous solution (previous biological treatment)	ESPA (Hydranautics)	8	-	326	99	[238]
Groundwater	BW30 (Filmtec)	13	1.5 × 10^−5^	15	94	[239]
Mining polluted groundwater	-	-	-	21	100	[240]
Potabilization inlet water	-	-	-	5	100	[241]
Previously NF treated landfill leachate	BW30 (Filmtec)	76	3.6 × 10^−6^	63	94	[242]

**Table 9:** Examples of application of nanofiltration for selenium removal from water.

Treated Water	Membrane	ΔP (bar)	Permeate Flux (m^3^/m^2^·s)	Initial [Se] (µg/L)	Removal (%)	Reference
Agricultural drainage water	Unidentified (Filmtec)	-	-	3000	95	[243]
Coal-fired power plant scrubber water	NF3A/PNF2 (SEPRO)	-	-	634	98.6	[148]
Synthetic aqueous solution	POSS-TFN (non-commercial)	10	1.5 × 10^−5^	100,000	97.4	[244]
Synthetic aqueous solution	UiO-66-TFN (non-commercial)	10	3.2 × 10^−5^	1,000,000	97.4	[236]
Synthetic aqueous solution	Zwitterionic copolymer-TFN (non-commercial)	10	2.4 × 10^−5^	1,000,000	99.9	[245]
Synthetic aqueous solution	Carbon quantum dots-TFN (non-commercial)	10	2.9 × 10^−5^	1,000,000	98.2	[246]
Synthetic aqueous solution	Polyamide intercalated membrane with biofunctionalized core shell composite (non-commercial)	0.5	1.2 × 10^−4^	100	98	[247]
Potabilization inlet water	NF1/NF2/NF20 (SEPRO)	14	3.9 × 10^−5^	400–2000	98	[237]

## Supplementary Materials

The following supporting information can be downloaded at: https://www.mdpi.com/article/10.3390/ijerph19105834/s1, Figure S1: Structure of the cluster *West-Bengal* in the second subperiod (2002–2011), Figure S2: Structure of the cluster *Glutathione-Peroxidase* in the second subperiod (2002–2011), Figure S3: Structure of the cluster *Speciation* in the second subperiod (2002–2011), Figure S4: Structure of the cluster *Plasma-Mass-Spectrometry* in the third subperiod (2012–2019), Figure S5: Structure of the cluster *Lead* in the third subperiod (2012–2019), Figure S6: Structure of the cluster *Soil* in the third subperiod (2012–2019), Figure S7: Structure of the cluster *Adsorption* in the third subperiod (2012–2019), Figure S8: Structure of the cluster *Supplementation* in the third subperiod (2012–2019), Figure S9: Structure of the cluster *Adsorption* in the fourth subperiod (2020–2021).
