# Supplementary material for: A Bibliometric Analysis of Research on Selenium in Drinking Water during the 1990–2021 Period: Treatment Options for Selenium Removal
Source: Int J Environ Res Public Health. 2022 May 11;19(10):5834. doi: 10.3390/ijerph19105834 (PMC9140891; doi:10.3390/ijerph19105834)
Supplement: Supplementary file 1 [file ijerph-19-05834-s001.zip › ijerph-1696852-supplementary.pdf]

Article

A Bibliometric Analysis of Research on Selenium in Drinking Water during the 1990–2021 Period: Treatment Options for Selenium Removal

Ricardo Abejón

Departamento de Ingeniería Química, Universidad de Santiago de Chile (USACH), Av. Libertador Bernardo O'Higgins 3363, Estación Central, Santiago 9170019, Chile; ricardo.abejon@usach.cl

Supplementary Material

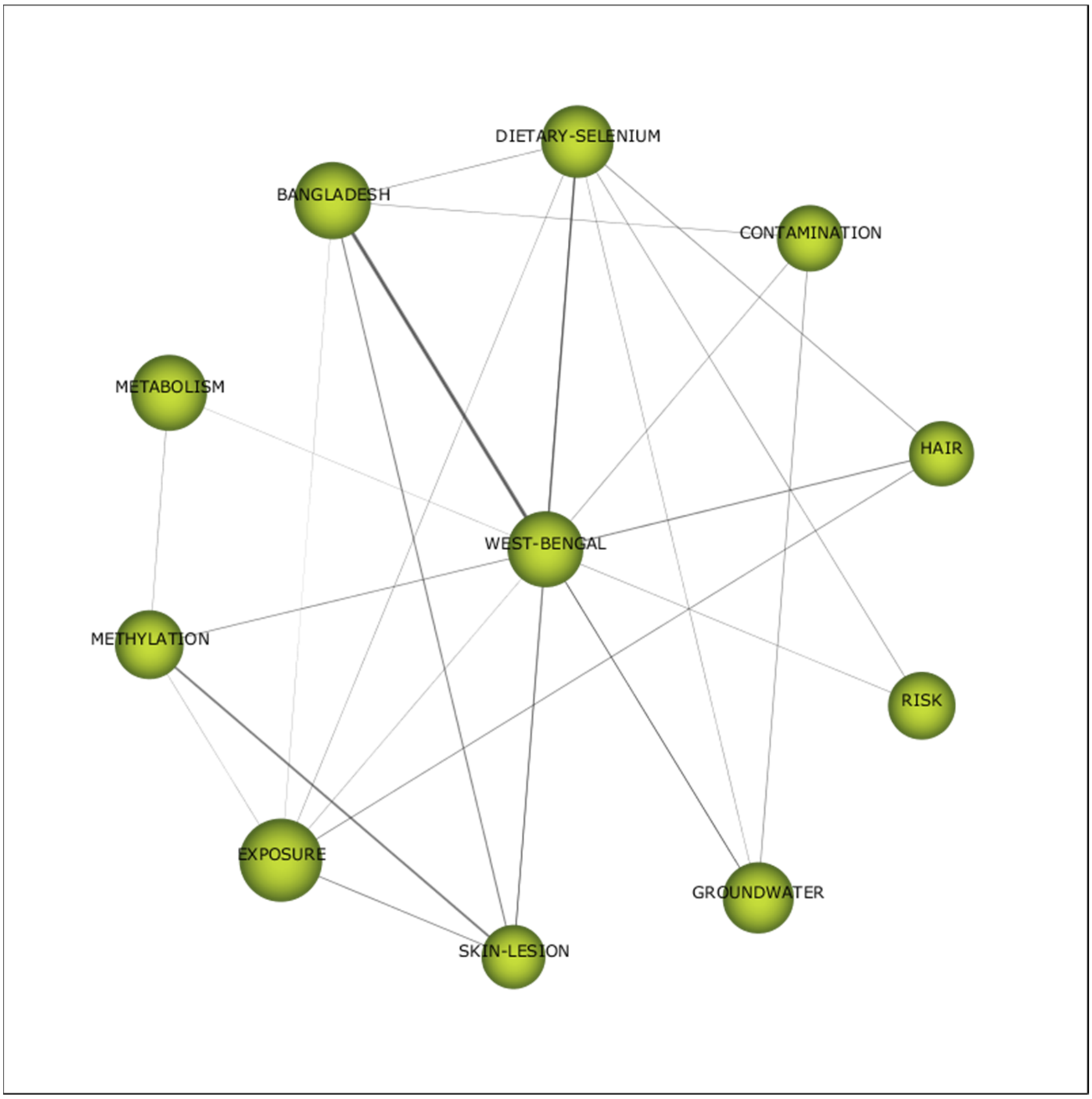

Figure S1. Structure of the cluster *West-Bengal* in the second subperiod (2002–2011).

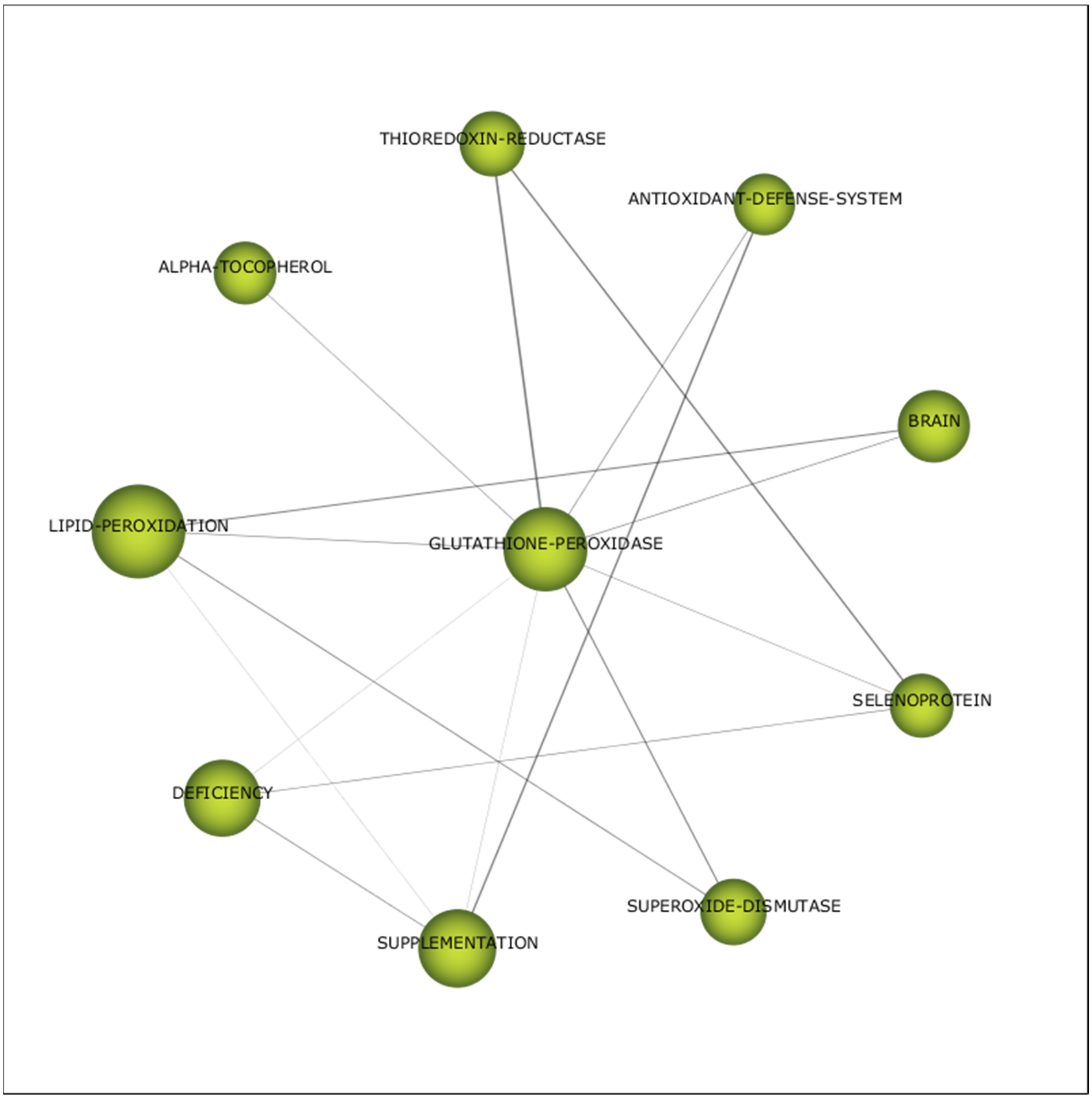

Figure S2. Structure of the cluster *Glutathione-Peroxidase* in the second subperiod (2002–2011).

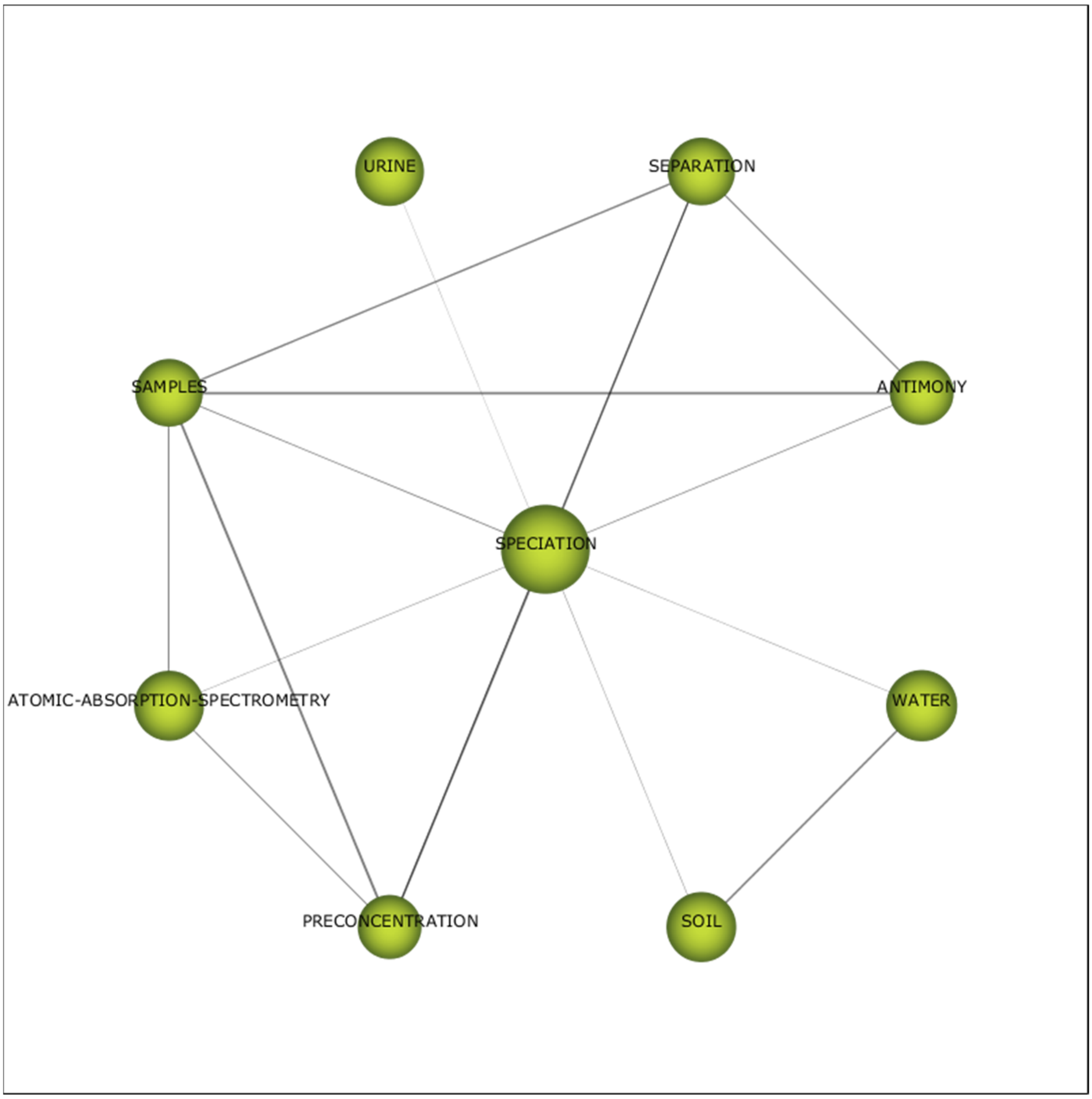

Figure S3. Structure of the cluster *Speciation* in the second subperiod (2002–2011).

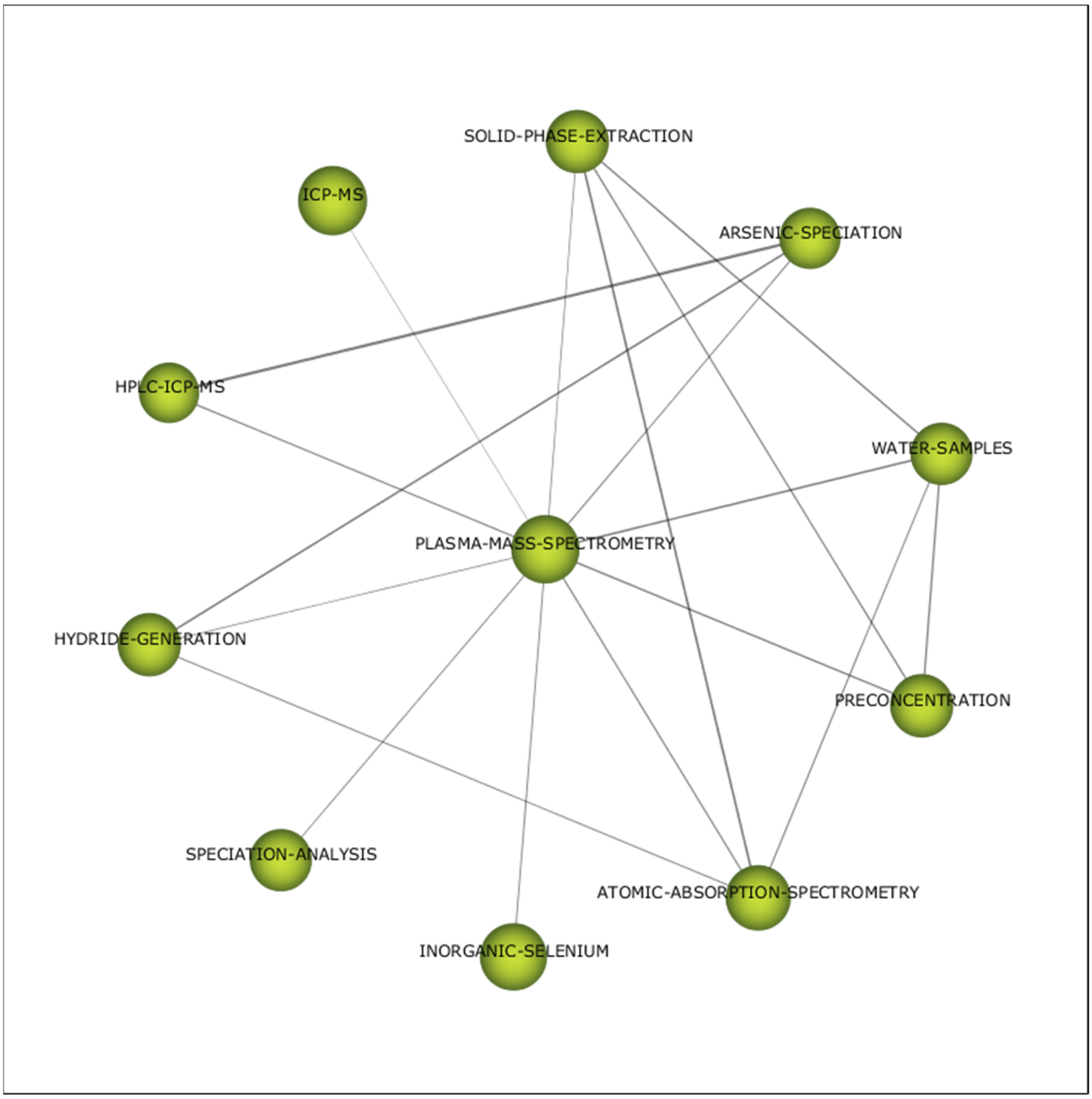

Figure S4. Structure of the cluster *Plasma-Mass-Spectrometry* in the third subperiod (2012–2019).

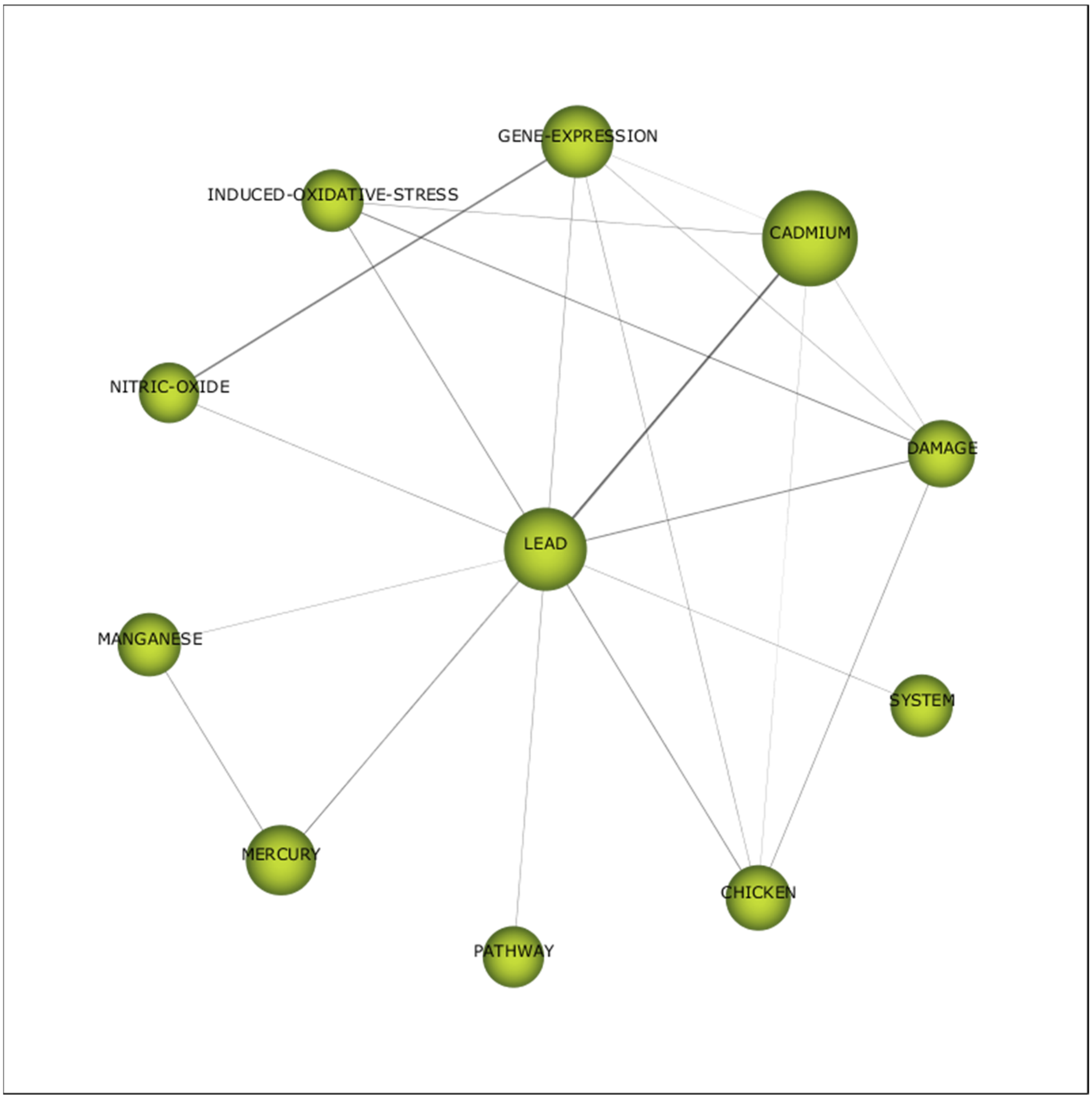

Figure S5. Structure of the cluster *Lead* in the third subperiod (2012–2019).

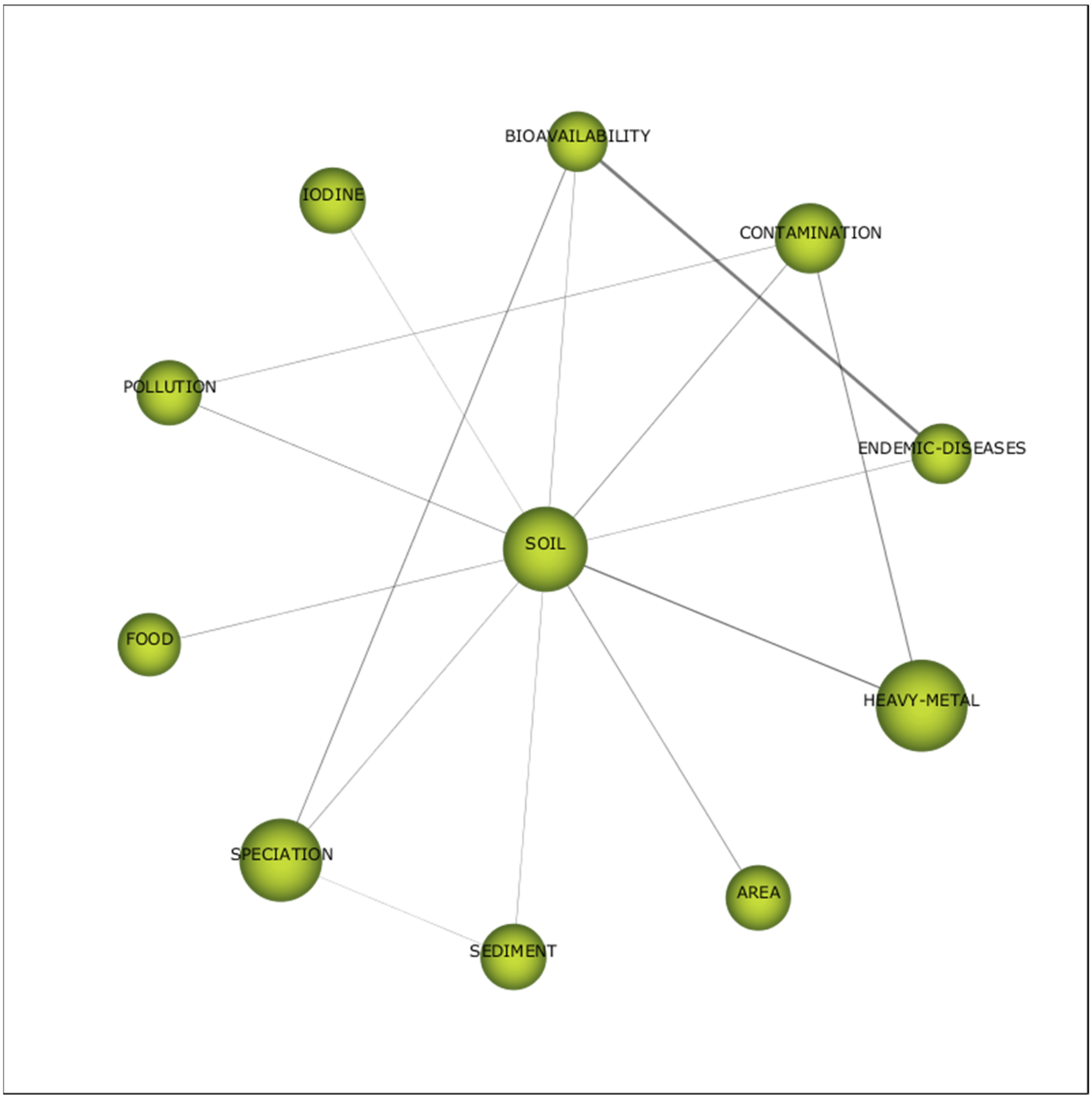

Figure S6. Structure of the cluster *Soil* in the third subperiod (2012–2019).

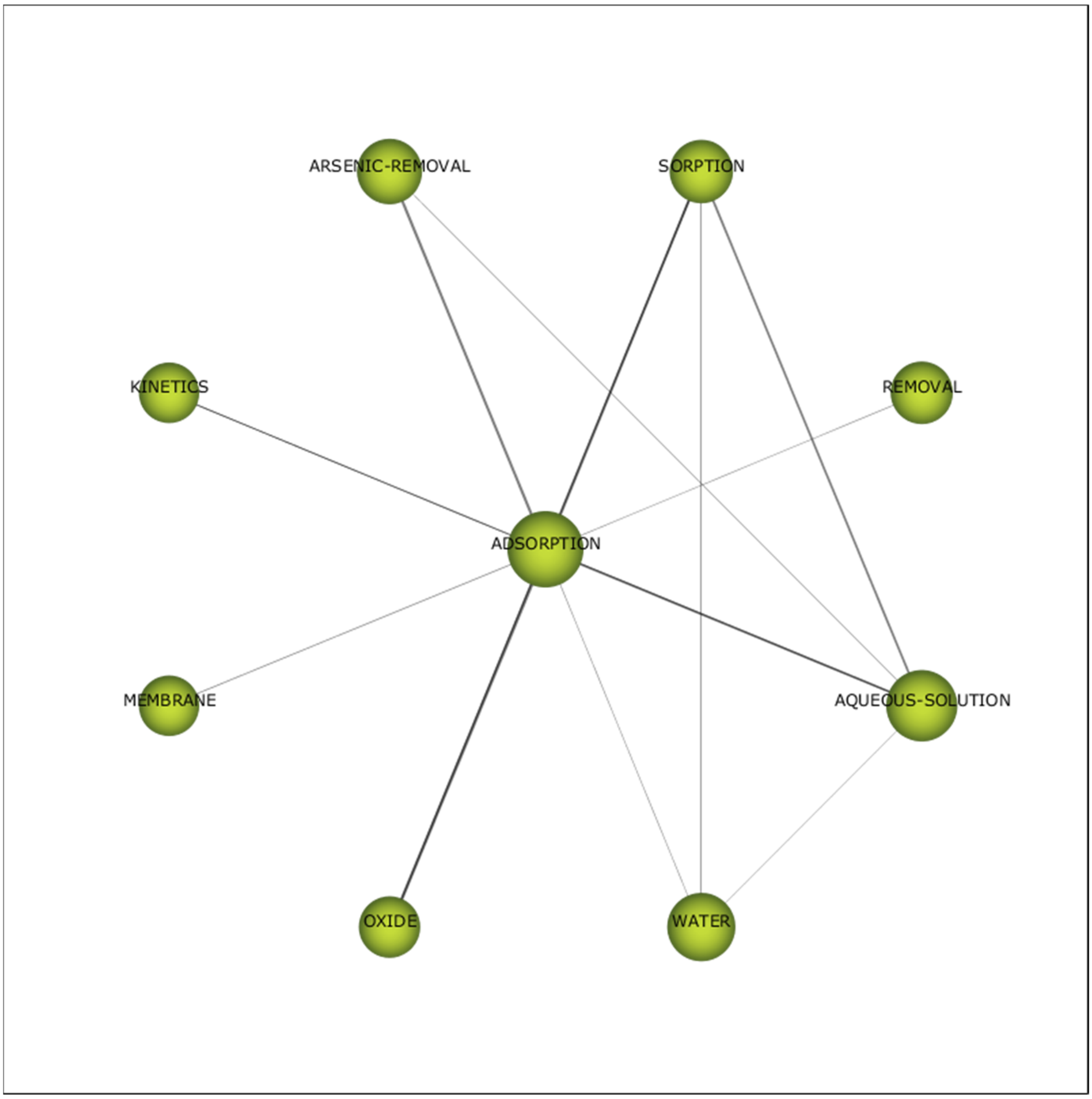

Figure S7. Structure of the cluster *Adsorption* in the third subperiod (2012–2019).

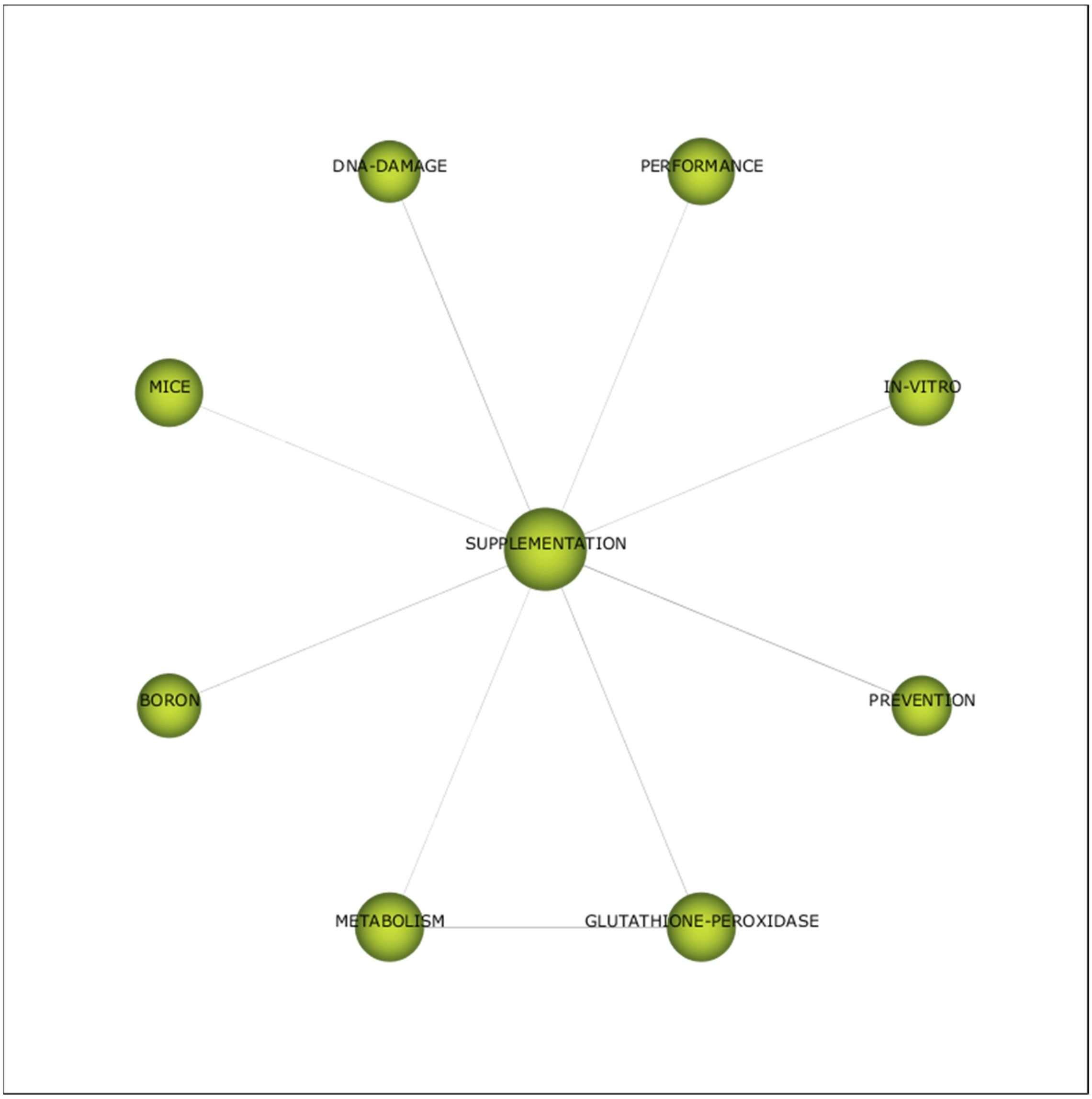

Figure S8. Structure of the cluster *Supplementation* in the third subperiod (2012–2019).

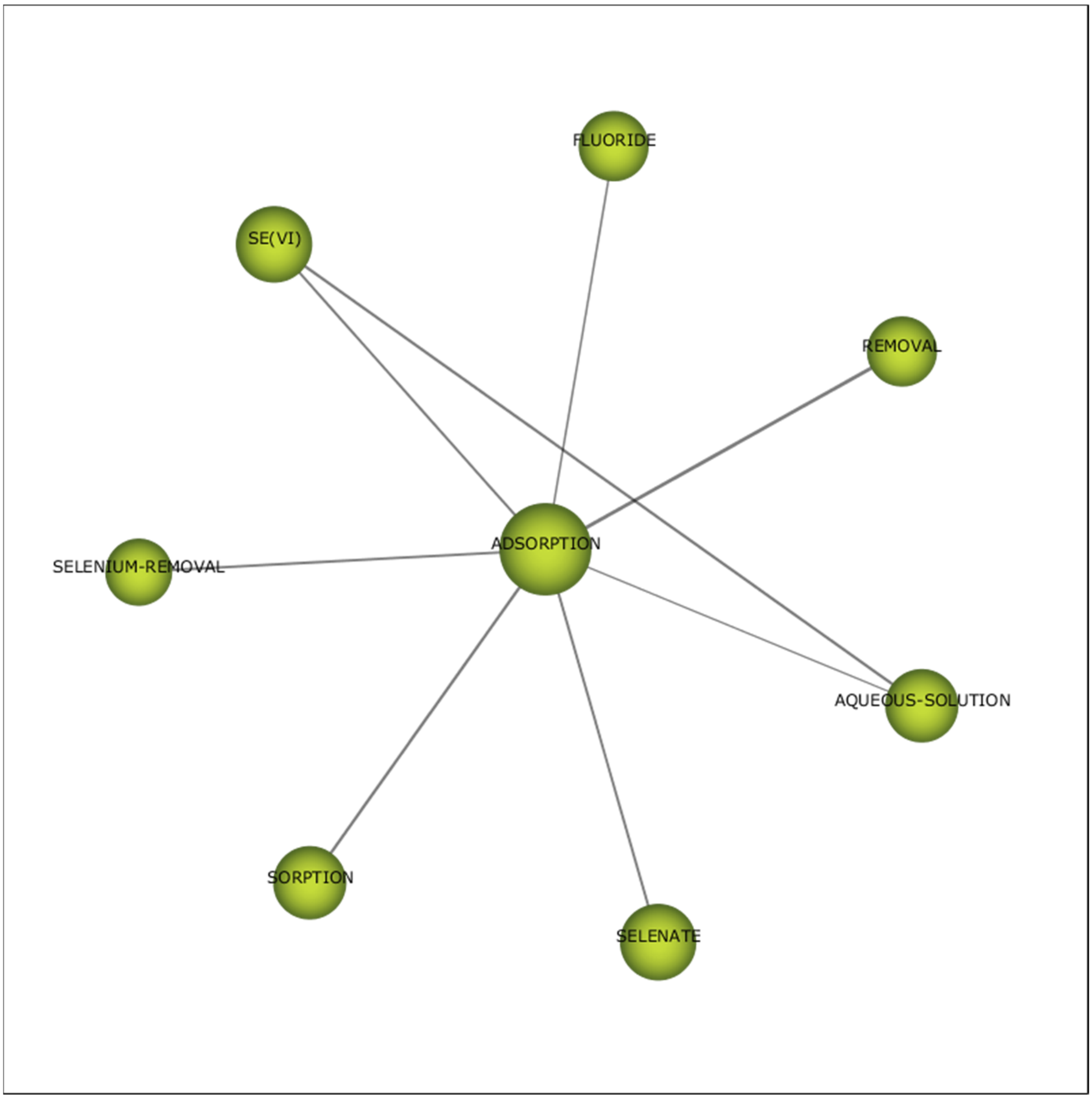

Figure S9. Structure of the cluster *Adsorption* in the fourth subperiod (2020–2021).
